# Risk-based innovations in cancer screening and diagnosis: a discrete choice experiment to explore priorities of the UK public

**DOI:** 10.1136/bmjopen-2024-093803

**Published:** 2025-05-31

**Authors:** Rebecca Dennison, Stephen Morris, Reanna J Clune, Stuart Wright, Jo Waller, Juliet Usher-Smith

**Affiliations:** 1 Department of Public Health and Primary Care, University of Cambridge, Cambridge, UK; 2 Health Services Research and Primary Care, The University of Manchester, Manchester, UK; 3 Wolfson Institute of Population Health, Queen Mary University of London, London, UK

**Keywords:** Mass Screening, Health policy, Risk Factors, Sensitivity and Specificity

## Abstract

**Abstract:**

**Objective:**

To understand the importance and potential impact on uptake of different attributes of risk-based innovations in the context of risk-stratified healthcare for cancer screening and symptomatic diagnosis.

**Design:**

The online survey comprised a discrete choice experiment (DCE) in which participants chose between two risk assessment options or to opt out of risk stratification. There were six attributes: test method, type (genetic or non-genetic), location, frequency, sensitivity and specificity. Participants were randomly allocated to consider the choice in an asymptomatic or symptomatic context.

**Setting:**

Members of the public in the UK.

**Participants:**

1202 participants completed the DCE.

**Outcome measures:**

Conditional logistic regression and latent class analysis informed modelling of predicted preferences for a range of innovations with different features.

**Results:**

Overall, participants preferred risk assessments over opting out and prioritised sensitivity, with test method and specificity also important. Genetic and non-invasive tests were favoured. With sensitivity and specificity of 80% or better, participants would be more likely to take up a risk assessment than not. Comparing the asymptomatic and symptomatic contexts, 65% and 73% of participants would be very likely to participate regardless of the innovation used, and 29% and 13% of participants might participate depending on the method, sensitivity and specificity. A minority showed strong dislike of risk-based innovations, particularly within screening.

**Conclusions:**

There are high levels of public support for risk-based innovations within risk-stratified cancer healthcare, especially for referral decision-making and using genetic and non-invasive tests. Optimising risk-based innovations is needed to engage those whose participation is contingent on test methods and performance metrics.

**STRENGTHS AND LIMITATIONS OF THIS STUDY**

- Splitting the sample into asymptomatic and symptomatic contexts enabled comparison of views towards different uses of risk assessments within early cancer diagnosis.
- Innovations were described according to a range of attributes, meaning that acceptability of a range of different technologies can be imputed, but preconceptions were not taken into account.
- The sample was relatively large and demographically representative of the UK population, although it may be biased towards those likely to engage in healthcare and healthcare research.

## Introduction

 With the burden of cancer projected to rise to 28.4 million cases globally in 2040, cancer prevention and early detection through screening and symptomatic diagnosis are a policy priority.^1^ For individuals, diagnosis at an earlier stage tends to be associated with a greater chance of positive treatment outcomes and long-term survival. For some cancers, the difference is stark: for example, 92% of patients with bowel cancer diagnosed at stage one survive for at least 5 years compared with just 10% of patients diagnosed at stage 4.^2^ Earlier stage diagnosis also has implications for healthcare systems where the cost of diagnosing and treating patients, particularly those with late-stage cancer, is growing.^3^ Furthermore, there is a need to offer the correct tests to the correct people to avoid unnecessary costs and harms, such as false-positive results and overdiagnosis.^4^

The implementation of novel technologies to estimate the risk of cancer and stratify healthcare has the potential to facilitate earlier stage diagnosis through multiple routes.^5 6^ For asymptomatic individuals, those at higher risk of cancer could be offered screening at an increased intensity (such as starting at a younger age, more frequently or with a lower threshold for investigation) and vice versa. For individuals presenting with non-specific symptoms, a risk classification could be used to support clinicians to decide which tests to use to investigate the symptoms and when they should take place that is urgent and more invasive/involved tests for those more likely to have cancer and vice versa.

Alongside more established genetic and phenotypic risk scores, technological developments are accelerating the field of early diagnosis.^5^ These range from the identification of cancer biomarkers to new materials for sensors within wearable devices and machine learning to process data.^57^,^12^ Furthermore, these new risk-based technologies have the potential to move risk prediction away from the traditional healthcare context. For instance, samples/data could be collected in the home or continuously over a period of time or through background analyses of routine medical records.^11 13^

One of the many requirements before the implementation of such technologies is demonstrated support from the public, as the target audience. Without public acceptability, there is the potential for mistrust of the technology and wider healthcare system.^14 15^ A growing body of recent research has demonstrated general support, although with some caveats, for risk-stratified cancer screening, particularly for increased screening among those at above average population risk.^16^ The risk stratification in these studies has typically included phenotypic or genetic risk rather than new and emerging technologies, with more comprehensive risk prediction strategies being preferred.^17^,^19^ Furthermore, the importance of logistical aspects of testing (eg, location and frequency of repeating tests) is unknown in this context.

Discrete choice experiments (DCEs) can be used to understand which aspects of healthcare services are most valuable to the target audience.^20 21^ This is achieved through a series of choice questions in which options for services are characterised by a set of attributes and levels and each time participants are asked to state their preference. This study aimed to quantify the relative importance and potential impact on uptake of different attributes of risk-based technologies and their application among members of the public. This was done in the context of risk-stratified healthcare for cancer screening and symptomatic diagnosis, without reference to a specific type of cancer or type of novel technology.

## Methods

### Patient and public involvement

Members of the public were involved from the beginning of the research process by expressing their support for the proposed study in the funding application. Four patient and public involvement (PPI) representatives contributed to study design and dissemination through discussions and written suggestions. These individuals had a range of characteristics across age, sex, history of cancer and prior PPI involvement. In particular, they considered the attributes to include in the DCE, how to explain these and other key concepts to the participants, how to phrase the questions and helped to develop lay summaries of the findings and implications.

### Survey design

This study involved a DCE embedded within an online survey hosted on Qualtrics XM (Washington, USA). A copy of the survey is available as described under data availability, alongside the dataset.^22^ Briefly, it began with an introduction to the study, a description of cancer screening and diagnostic concepts and an explanation of the attributes and levels included in the DCE. This was followed by three questions to examine comprehension of the concepts and ability to interpret the information presented in the question.

The main part of the survey was the DCE. In this section, each participant had to answer nine questions, which appeared in a random order. Each question included two options for risk assessments that would inform risk stratification, plus an opt-out option (described as there being no assessment of cancer risk and so everyone would be screened according to the same policy or referred based on clinical judgement alone). In each question, participants were asked which option they thought was most acceptable. Participants were randomly allocated to answer questions either in the context of cancer screening (asymptomatic context cohort) or of referral for investigation of symptoms that could be cancer (symptomatic context cohort). An example question is detailed in online supplemental table 1.

The attributes included within each question were (1) the method of risk assessment (how the data for the risk assessment are collected); (2) type of risk assessment (genetic data or non-genetic); (3) location of risk assessment (where data collection takes place); (4) frequency of risk assessment (how often data collection needs to be repeated); (5) the number of people whose risk of cancer would be overestimated, a measure of specificity; and (6) the number of people whose risk of cancer would be underestimated, a measure of sensitivity. These attributes were informed by reviews of the literature, our qualitative research that formed part of the same grant and discussion with experts in DCE design and PPI representatives. The qualitative research was a set of three community juries (24 participants in total) and think-aloud interviews (21 participants) in which members of the public considered six different innovative approaches to be acceptable to assess the risk of cancer.^23 24^ We considered what we learnt in these studies about which characteristics of risk assessments most impact public opinions. For example, we found that test accuracy was essential to the public so included sensitivity and specificity attributes; effort/burden to take part was also important so we included attributes for test location and frequency. The levels, shown in table 1, reflected a plausible and clinically relevant range while also avoiding extreme values to limit grounding effects.

**Table 1:** Attributes and levels used in the discrete choice experiment

Attribute	Levels	Explanation given to participants
Method of risk assessment	1. Questionnaire or data access	Cancer risk can be estimated using data from the person’s existing health records (with their permission), or they could do a questionnaire to provide additional information.
	2. Blood test	This will require the person to provide a blood sample. It will be sent to a laboratory for analysis of certain biomarkers (which are signals of what is going on in the body).
	3. Non-invasive test	This quick and easy test may include providing a sample of urine, stool or saliva. The sample will be analysed for certain biomarkers (which are signals of what is going on in the body).
	4. Wearable device	A device like a smartwatch, patch or sensor is worn to continuously monitor factors such as sleep patterns, heart rate and temperature or to monitor biomarker levels (which are signals of what is going on in the body).
Type of risk assessment	1. Genetic	A person’s genes or DNA are analysed to estimate their cancer risk.
	2. Non-genetic	Data other than a person’s genes or DNA are analysed to estimate their cancer risk.
Location of risk assessment	1. Home	The test is carried out by the person, in their own home.
	2. Community clinic/pharmacy	The person would go to a community clinic (in a supermarket, for example) or pharmacy to have the test carried out.
	3. General practice	The person would go to their GP or nurse to have the test carried out.
	4. Hospital	The person would go to the hospital to have the test carried out.
Frequency of risk assessment	1. One-off single event	They would only do the test one time ever.
	2. Once every 5 years	They would do the test once every 5 years.
	Once every year	They would do the test once a year.
	3. Continuously for a 2 week period	They would wear the device continuously over a 2 week period.
	Constantly	They would wear the device continuously over a prolonged period of time.
Accuracy – risk of cancer is overestimated (specificity)	5 (95%) 10 (90%) 15 (85%) 20 (80%) …out of every 100 people who have a risk assessment	# out of every 100 people who have a risk assessment are told they are at high risk of cancer when actually they are not. This means that they might be offered more screening or more diagnostic tests than they should be, based on their actual cancer risk. The tests may cause more harm than benefit.
Accuracy—risk of cancer is underestimated (sensitivity)	5 (95%) 10 (90%) 15 (85%) 20 (80%) …out of every 100 people who have a risk assessment	# out of every 100 people who have a risk assessment are told they are at low risk of cancer when actually their risk is higher. This means they might be offered less screening or fewer diagnostic tests than they should be, based on their actual cancer risk. This might mean a cancer is diagnosed later than it could have been if they had been offered more tests.

GP, general practitioner.

A d-efficient design was used to combine the levels for 18 questions using the Stata 15 (StataCorp, Texas, USA) command -dcreate-. Illogical or nonsense combinations of levels were removed from all possible options; for example, genetic risk assessments could only be paired with blood or non-invasive tests (ie, a saliva sample). The questions were then split into two sets of nine questions using -blockdes-, which were randomly assigned to participants 1:1 in the asymptomatic and symptomatic scenarios. The combination of levels is presented in online supplemental table 2.

At the end of the survey, participants were asked to provide grouped demographic information including age group, sex, ethnicity, socioeconomic status decile and cancer screening history, and their perceptions of cancer and screening using validated questions where available.

Ethical approval was obtained from the University of Cambridge Humanities and Social Sciences Research Ethics Committee (reference 23.342).

### Participants and recruitment

1200 individuals resident in the UK were recruited through Prolific (London, UK). Prolific is an online-only research recruitment platform with a pool of registered participants. It does not focus on health research and has been found to result in the collection of higher-quality data than comparable platforms.^25^ The sample was broadly representative of the population in accordance with Prolific’s capabilities. This was achieved by first recruiting 750 participants in proportions representative of the UK population with regard to age, sex and ethnicity, and then 450 participants of self-reported socioeconomic status 1–3 (where one is the lowest decile) balanced by sex.

Participants first saw a brief overview of the study. If interested, they could access the participant information sheet and then give informed consent online before entering the main survey. This included confirming that they understood their participation is voluntary, they can withdraw consent at any time and their responses will become publicly available in an anonymous, online database at the end of the study. They were paid £2.50 for taking part.

### Analysis

We used Stata 15 (StataCorp, Texas, USA) and Microsoft Excel (Microsoft Corporation, Washington, USA) for the analyses. Descriptive statistics were used to summarise the characteristics of the participants and their beliefs about cancer. We also counted the frequency at which participants opted out of risk assessments. The asymptomatic and symptomatic context cohorts were analysed separately.

The main analysis used conditional logistic regression models (fixed effects logit; -clogit- command) to indicate participants’ preferences for different aspects of risk assessments.^21^ The accuracy attributes were coded as continuous variables, and all others were dummy coded. These were found to be the models that best fitted the data according to the Akaike and Bayesian Information Criteria (AIC and BIC; online supplemental table 3A). X^2^ tests compared preferences of those who completed the DCE in the asymptomatic context with those who completed it in the symptomatic context. We also ran two sensitivity analyses: first, excluding those who showed poor attention by completing the survey in the fastest 5% (<7.5 min) or always selecting Option one or two; second, excluding those who showed lower understanding of the DCE by not answering the three comprehension questions correctly.

Latent class analysis was then used to divide the participants into groups with similar preferences and priorities for risk assessments. We then examined whether the classes could be described by the participants’ characteristics (eg, whether female participants were statistically more likely to be in one class than another). The optimal number of groups, analysis seed and associations with binary characteristics were identified by examining the BIC (online supplemental table 3B–D).

Additionally, we used the coefficients generated in the main analysis in several calculations.^26^ In these, accuracy attributes were converted to sensitivity and specificity of the risk assessment (sensitivity = 100 − underestimated risk percentage; specificity = 100 − overestimated risk percentage). We calculated the relative importance of each attribute by comparing the difference in the coefficients of the most and least preferred level of each attribute (or, for continuous attributes, the difference in the coefficients multiplied by the most and least preferred level). We also calculated the trade-offs participants were willing to make in terms of sensitivity and specificity using marginal rates of substitution, with CIs calculated using the Delta method (-nlcom- command).^27^ Additionally, we modelled the probability of preferring five different risk-based innovations (with details of the levels modelled included in the figure legends, below). In this last analysis, no risk assessment was used as the comparator (reference). There is no unique best way to analyse opt-out data collected from DCEs.^28^ We set the categorical variables to zero to indicate no method, type, location or frequency of risk assessment. We fixed the value of the accuracy attributes at 12.5 out of every 100 people who have a risk assessment with over- and underestimated risk. This is the mean of the levels these attributes could take (range 5 to 20). We considered setting the value to zero, but this would have implied a perfect risk classification, which is nonsensical in the case of no risk assessment. When the accuracy attributes were fixed at different levels, the trends between probabilities did not change, but the value of the predicted probability did change (online supplemental figure 1).

## Results

### Participant characteristics

The survey was live for approximately 70 hours between 21 and 24 November 2023. 1288 individuals registered with the recruitment platform accessed the survey. 70 individuals withdrew by returning their submissions, 14 timed out and 1204 completed the survey. Two were excluded because they completed the survey exceptionally quickly (in 3 min), and their responses suggested that they had not considered their answers.

Median time to complete the survey was 15 min and 9 s (IQR 11 min and 22 s to 21 min and 36 s). All three of the questions designed to test comprehension of the table of risk assessment options, completed before the DCE exercise, were answered correctly by 74% (883 participants), with an average 90% of participants answering each question correctly (online supplemental table 4).

601 participants were randomised to the asymptomatic context cohort and 601 participants were randomised to the symptomatic context cohort. Table 2 and online supplemental table 5 show that the participants’ demographic characteristics and thoughts and beliefs about cancer and screening were similar between cohorts; the only statistically significant difference was that more participants in the asymptomatic context cohort had a personal history of cancer. Reflecting the representative sampling strategy, 85.8% of the total sample reported their ethnicity as White (1031 participants) and 26.7% considered their socioeconomic status to be in the lowest three deciles (321 participants).

**Table 2:** Participant demographics

Category (p value for difference*)	Asymptomatic context cohort	Symptomatic context cohort	Total (%)
Total *N*	601	601	1202 (100)
Age (years) (p=0.987)			
18–29	140	146	286 (23.8)
30–39	143	135	278 (23.1)
40–49	106	111	217 (18.1)
50–59	100	97	197 (16.4)
60–69	86	88	174 (14.5)
≥70	26	24	50 (4.2)
Sex† (p=0.414)			
Female	318	295	613 (51.0)
Male	282	305	587 (48.8)
Other	1	1	2 (0.2)
Simplified ethnicity (p=0.921)			
Asian	43	38	81 (6.7)
Black	24	23	47 (3.9)
Mixed	13	16	29 (2.4)
White	515	516	1031 (85.8)
Other	6	8	14 (1.2)
Self-reported socioeconomic status decile (p=0.200)			
1–3 (lowest)	168	153	321 (26.7)
4–5	195	212	407 (33.9)
6–7	213	198	411 (34.2)
8–10 (highest)	25	38	63 (5.2)
Educational level (p=0.780)			
Not completed A levels, further education or equivalent	100	94	194 (16.1)
Competed A levels, further education or equivalent	191	188	379 (31.5)
Completed a bachelor’s degree	216	212	428 (35.6)
Completed a postgraduate degree	94	107	201 (16.7)
Location (p=0.782)			
England	511	498	1009 (83.9)
Northern Ireland	14	15	29 (2.4)
Scotland	52	60	112 (9.3)
Wales	24	28	52 (4.3)
Smoking status (p=0.432)			
Never smoked	358	351	709 (59.0)
Used to smoke	178	195	373 (31.0)
Current smoker	65	55	120 (10.0)
Weight (p=0.347)			
Underweight	31	21	52 (4.3)
About the right weight	314	314	628 (52.3)
Overweight	256	266	522 (43.4)
Personal history of cancer (p=0.016)			
Yes	35	18	53 (4.4)
No	562	582	1144 (95.2)
Not reported	4	1	5 (0.4)
Previously have completed cancer screening (p=0.646)			
Yes	281	265	546 (45.4)
No (chose not to)	37	38	75 (6.2)
No (never been invited)	280	295	575 (47.8)
Not reported	3	3	6 (0.5)

* p value for difference between asymptomatic and symptomatic context cohorts based on X2 tests.

† 19 (1.6%) participants reported that their gender was not the same as the sex that they were assigned at birth. Of these, nine participants identified as non-binary.

Ease of completing the DCE was similar for the asymptomatic and symptomatic context cohorts. Most participants found it difficult (59.2%, 712/1,202 participants), as shown in online supplemental figure 2.

### Preferences for risk stratification

The coefficients for the constant (no risk assessment nor stratification) in the main analyses were negative, indicating preferences for risk assessments (−0.679 (p<0.001) and −0.829 (p<0.001), for asymptomatic and symptomatic context cohorts, respectively; table 3).

**Table 3:** Participants’ preferences for risk assessments and the relative importance of each attribute in asymptomatic and symptomatic context cohorts (conditional logistic regression analysis)

	Asymptomatic context cohort	Symptomatic context cohort	P value for difference between cohorts
*N* participants	601	601	**<0.001** (between models as a whole)
*N* observations	16 227	16 227	
Pseudo R^2^	0.0975	0.2118	
Constant			
No risk assessment	**−0.679 (−0.823 to −0.536**)	**−0.829 (−0.986 to −0.672**)	0.165
Method of risk assessment			
Questionnaire or data access	Reference	Reference	
Blood test	**0.321 (0.194 to 0.448**)	**0.956 (0.822 to 1.090**)	**<0.001**
Non-invasive test	**0.316 (0.207 to 0.425**)	**0.717 (0.602 to 0.832**)	**<0.001**
Wearable device	**−0.184 (−0.353 to −0.014**)	**0.273 (0.101 to 0.444**)	**<0.001**
Type of risk assessment			
Non-genetic	Reference	Reference	
Genetic	−0.007 (−0.153 to 0.138)	0.147 (−0.011 to 0.305)	0.163
Location of risk assessment			
Home	Reference	Reference	
Community clinic/pharmacy	−0.029 (−0.141 to 0.084)	0.106 (−0.011 to 0.223)	0.107
General practice	−0.097 (−0.213 to 0.018)	−0.009 (−0.133 to 0.114)	0.311
Hospital	**−0.221 (−0.330 to −0.113**)	−0.029 (−0.136 to 0.078)	**0.014**
Frequency of risk assessment			
One-off single event	Reference	Reference	
Once every 5 years	−0.002 (−0.137 to 0.133)	0.006 (−0.139 to 0.150)	0.941
Once every year	−0.056 (−0.172 to 0.059)	−0.001 (−0.122 to 0.120)	0.512
Continuously for 2 weeks	0.205 (−0.043 to 0.454)	−0.032 (−0.283 to 0.219)	0.185
Constantly	−0.034 (−0.198 to 0.130)	0.099 (−0.073 to 0.270)	0.269
Accuracy			
Specificity	**0.042 (0.035 to 0.049**)	**0.048 (0.040 to 0.055**)	0.262
Sensitivity	**0.059 (0.053 to 0.066**)	**0.081 (0.074 to 0.088**)	**<0.001**

Results where p<0.05 are highlighted in bold.

Accuracy levels were presented as number of people out of every 100 people who have a risk assessment whose risk will be overestimated [specificity]/underestimated [sensitivity].

Positive coefficients indicate a preference for the specified level compared to the reference; negative coefficients indicate a preference for the reference compared to the specified level.

**Figure 1:**
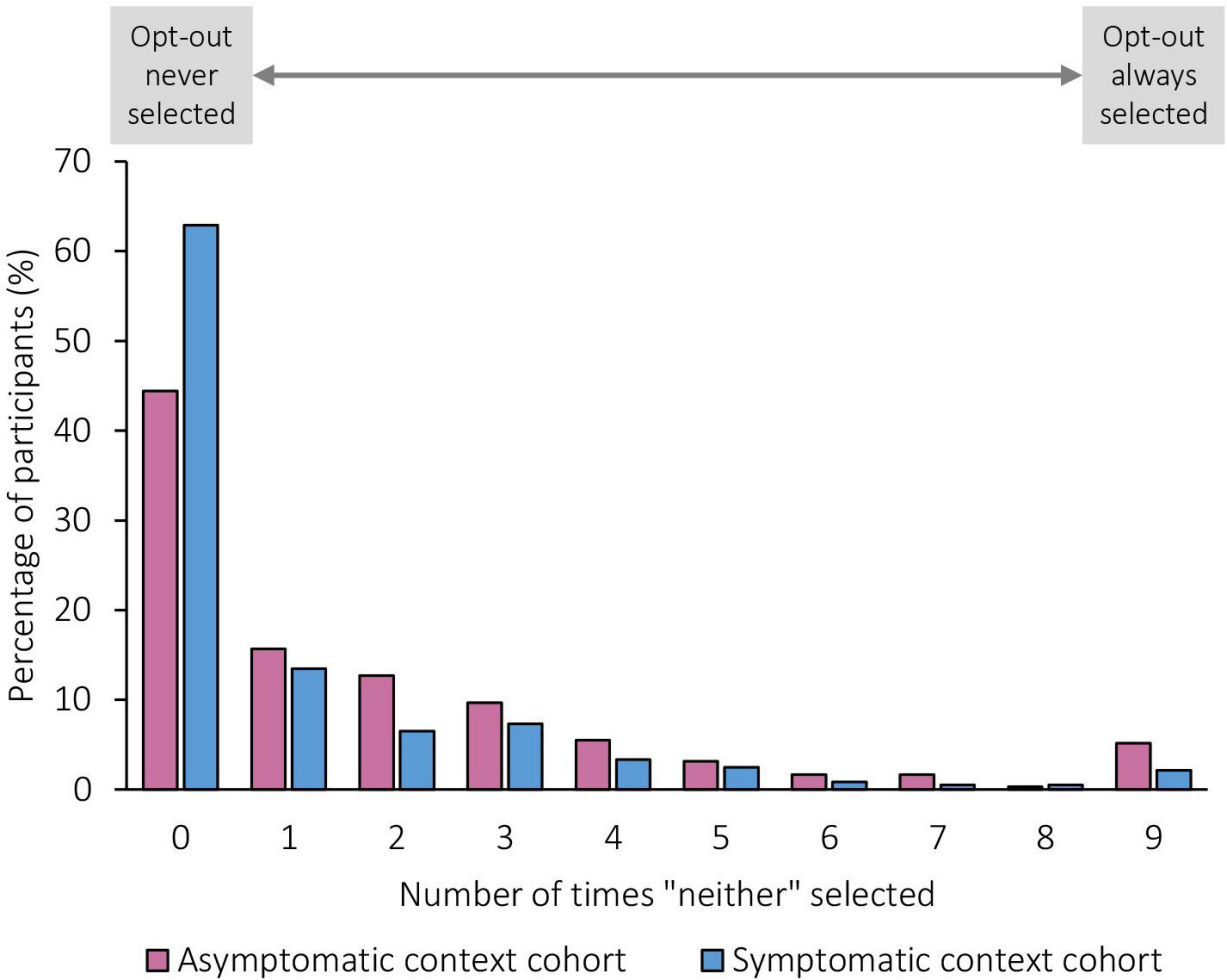
Frequency at which participants opted not to estimate risk of cancer across nine questions.

Consistent with this, participants chose one of the risk assessment and stratification options in most instances. The “neither” option, indicating a preference not to estimate the risk of cancer, was selected 19.9% of the time in the asymptomatic context cohort (1077 out of a total 5409 responses) and 11.8% of the time in the symptomatic context cohort (638 out of a total 5409 responses); p value for difference ≤0.001. Just over half (53.6%; n=644/1,202) participants never selected the “neither” option, while 3.7% (n=44/1,202) participants selected “neither” in response to all questions. Frequency of selecting “neither” is summarised in figure 1.

#### Preferences for different aspects of risk assessments

The method of risk assessment and accuracy significantly affected preferences in both cohorts (table 3). Blood tests and non-invasive tests were preferred over questionnaires or data access. Compared with questionnaires or data access, wearable devices were preferred in the symptomatic context, but less preferred in the asymptomatic context. In both contexts, participants preferred risk assessments that more accurately estimated the risk of cancer. The importance of specificity was similar in the two contexts, with coefficients of 0.042 and 0.048 (p=0.262 for difference between cohorts). It was more important to have higher sensitivity for people with symptoms than in those without symptoms, with coefficients of 0.081 and 0.059, respectively (p<0.001 for difference between cohorts). There were no differences between context cohorts in preference for whether a test was genetic or non-genetic, location or frequency of repeating the test, except that it was seen as unfavourable for asymptomatic people to attend the hospital compared with doing a home-based test. Participants in the asymptomatic and symptomatic contexts had different preferences across the models as a whole (p<0.001).

The sign of significant coefficients in the sensitivity analyses (online supplemental table 6) was consistent with those in the main analyses. Any differences in the magnitude of the coefficients were small. One notable exception was that participants who showed better comprehension preferred genetic tests over non-genetic tests in the symptomatic context. The magnitude of the coefficients for sensitivity and specificity was also always slightly larger in the sensitivity analyses.

#### Relative importance of different attributes of risk assessments

**Figure 2:**
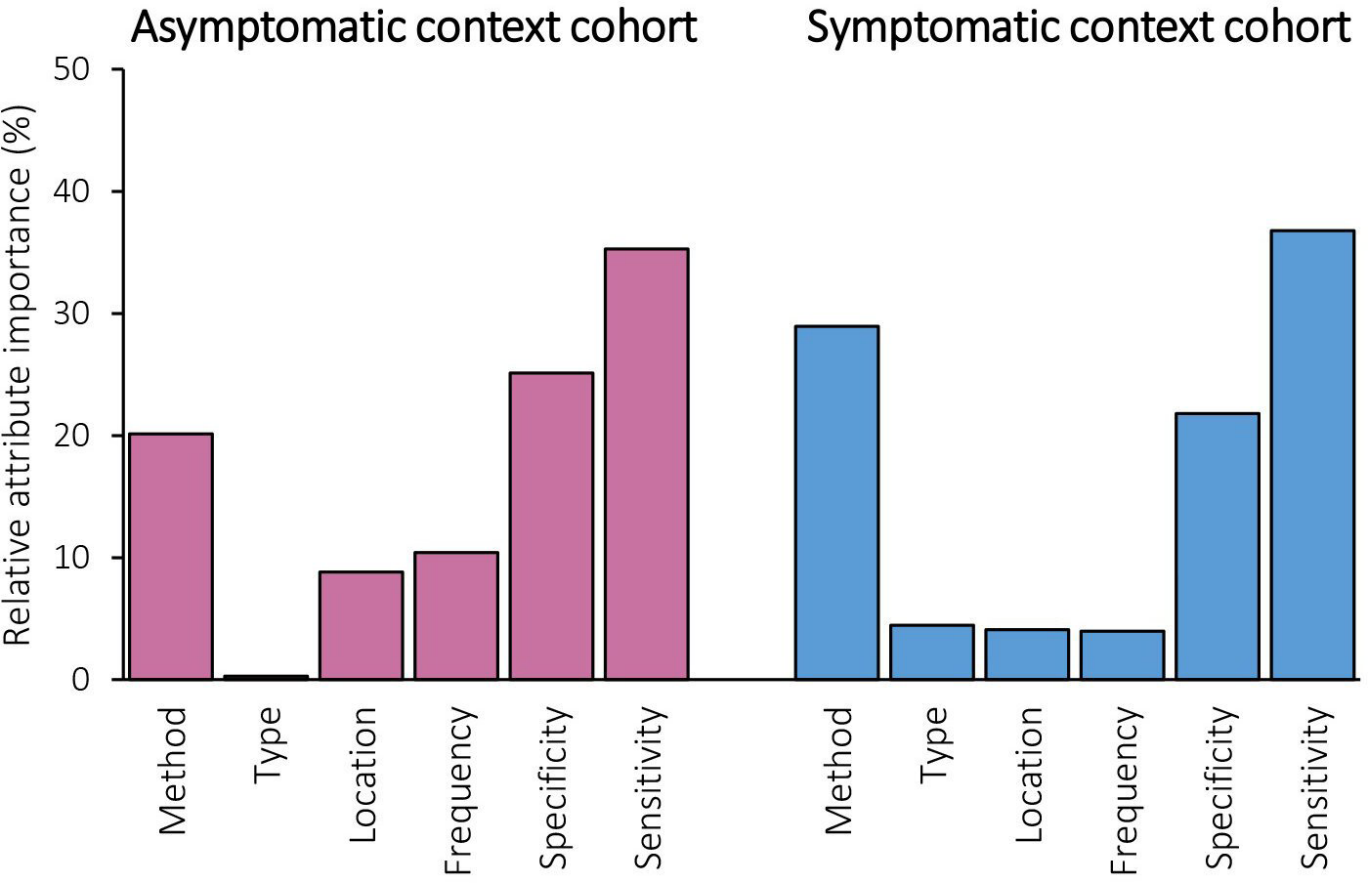
Relative attribute importance.

In both cohorts, sensitivity was the most important attribute (relative importance of 35.3% for asymptomatic and 36.8% for symptomatic; figure 2). Specificity was more important than the method used in the asymptomatic context cohort (25.1% for specificity vs 20.1% for method), whereas the method was more important than specificity in the symptomatic context (21.8% for specificity vs 28.9% for method). Whether it was a genetic or non-genetic test, the location and frequency were the three least important attributes in both contexts.

#### Willingness to trade-off sensitivity and specificity of risk assessments

**Figure 3:**
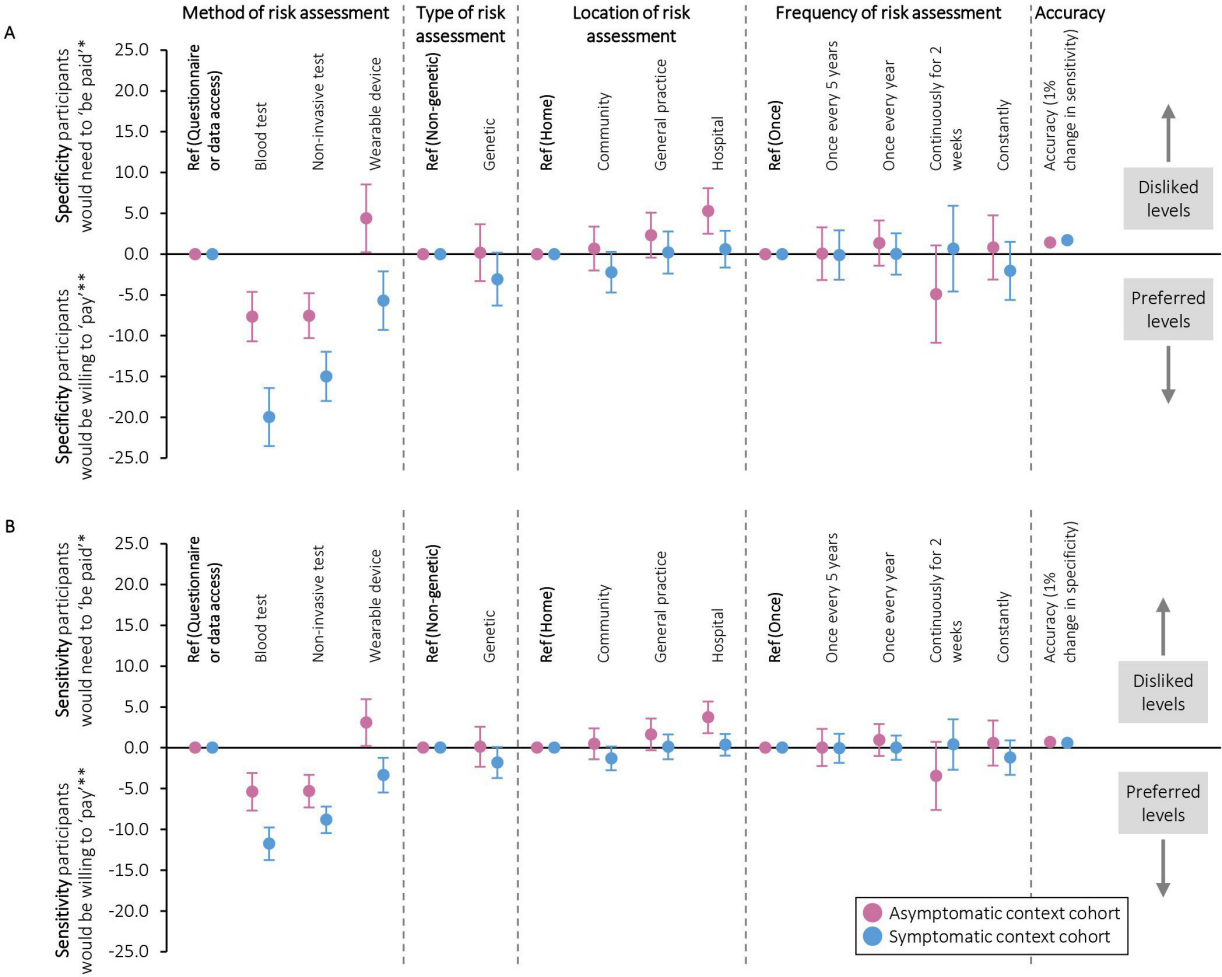
Trade-offs that participants were willing to make between (a) specificity and (b) sensitivity and different aspects of cancer risk assessments (conditional logistic regression analysis). *Increase in risk assessment specificity/sensitivity that participants would need to ‘be paid’ in order to accept their less preferred options compared with the reference. **Decrease in risk assessment specificity/sensitivity that participants would be willing to ‘pay’ for their preferred options compared with the reference.

Figure 3 shows participants’ willingness to trade-off sensitivity and specificity in order to use their preferred risk assessments. For example, they were willing to forego a 5% decrease in sensitivity in the asymptomatic context and a 12% decrease in sensitivity in the symptomatic context in order to have blood tests rather than data-based risk assessments (95% CIs −8 to −3% and −10 to −14%, respectively). When trading off sensitivity and specificity, they were willing to accept a 1% decrease in sensitivity for a 1.4% increase in specificity in the asymptomatic context (95% CI 1.3 to 1.6%), and a 1% decrease in sensitivity for a 1.7% increase in specificity in the symptomatic context (95% CI 1.5 to 1.8%).

#### Modelled preferences for different risk assessments

**Figure 4:**
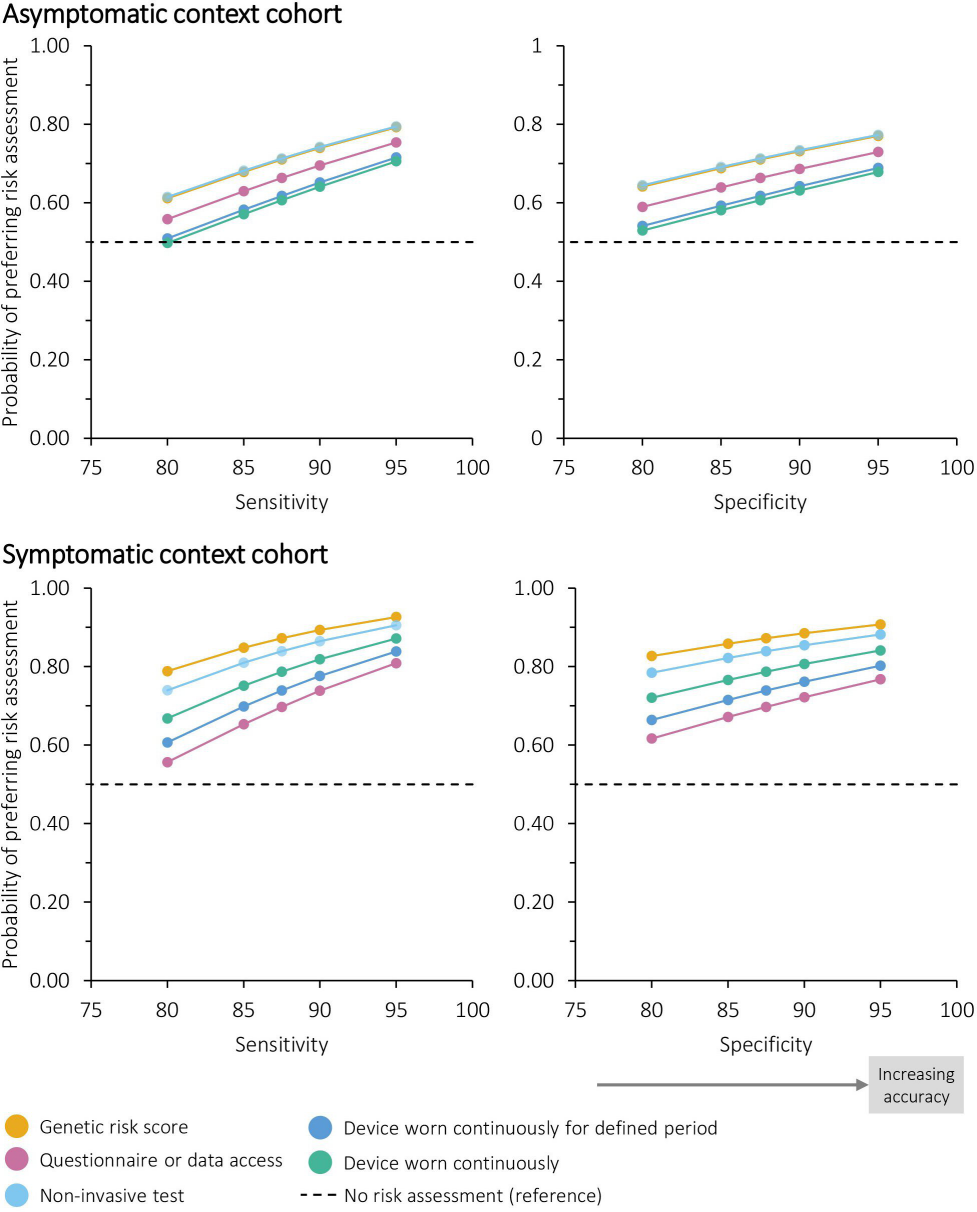
Probability of preferring different risk assessments at differing levels of accuracy (conditional logistic regression analysis). Each example of risk-based innovation and the reference was modelled as (method, type, location, frequency, sensitivity and specificity): (1) genetic risk score: blood test, genetic, general practice, one-off single event, 95% or as specified. (2) Questionnaire or data access: questionnaire or data access, non-genetic, home, once every 5 years, 95% or as specified. (3) Non-invasive test (saliva or blood test): non-invasive test, non-genetic, community, once every year, 95% or as specified. (4) Device worn continuously for defined period: wearable device, non-genetic, hospital, continuously for a 2 week period, 95% or as specified. (5) Device worn continuously: wearable device, non-genetic, community, constantly, 95% or as specified. (6) No risk assessment (reference): none, none, none, none, 87.5% (plus opt-out coefficient).

Figure 4 illustrates the relative preference for five plausible examples of risk-based innovations, defined using the method, type, location and frequency attributes and modelled with varying accuracy. From this, the order of preference in the asymptomatic context cohort was genetic tests and non-invasive tests, data-based risk assessments and then wearable devices. The order of preference in the symptomatic context cohort was genetic tests, non-invasive tests, devices worn continuously, devices worn for a defined period and then data-based risk assessments.

For the same sensitivity and specificity, genetic and non-invasive tests would be equally favoured for screening in the asymptomatic context. In order for the probability of preference over no risk assessment to be 75%, sensitivity of genetic and non-invasive tests would need to be approximately 87% when specificity is 87.5%. Risk assessments based on questionnaire data or medical records and the two options for wearable devices required higher sensitivity and specificity for the same probability of preference.

In the symptomatic context, all risk assessments had a greater than 55% probability of being preferred over no risk assessment at the sensitivity and specificity modelled. With sensitivity fixed at 87.5%, genetic risk scores needed a specificity better than 84% to have an approximately 85% probability of being preferred over no risk assessment, non-invasive tests needed 89% and continuously worn devices needed 95%. With specificity fixed at 87.5%, genetic risk scores needed a sensitivity better than 85% in order for approximately 85% probability of being preferred over no risk assessment, non-invasive tests needed 89% and continuously worn devices needed 93%.

#### Preference heterogeneity

**Figure 5:**
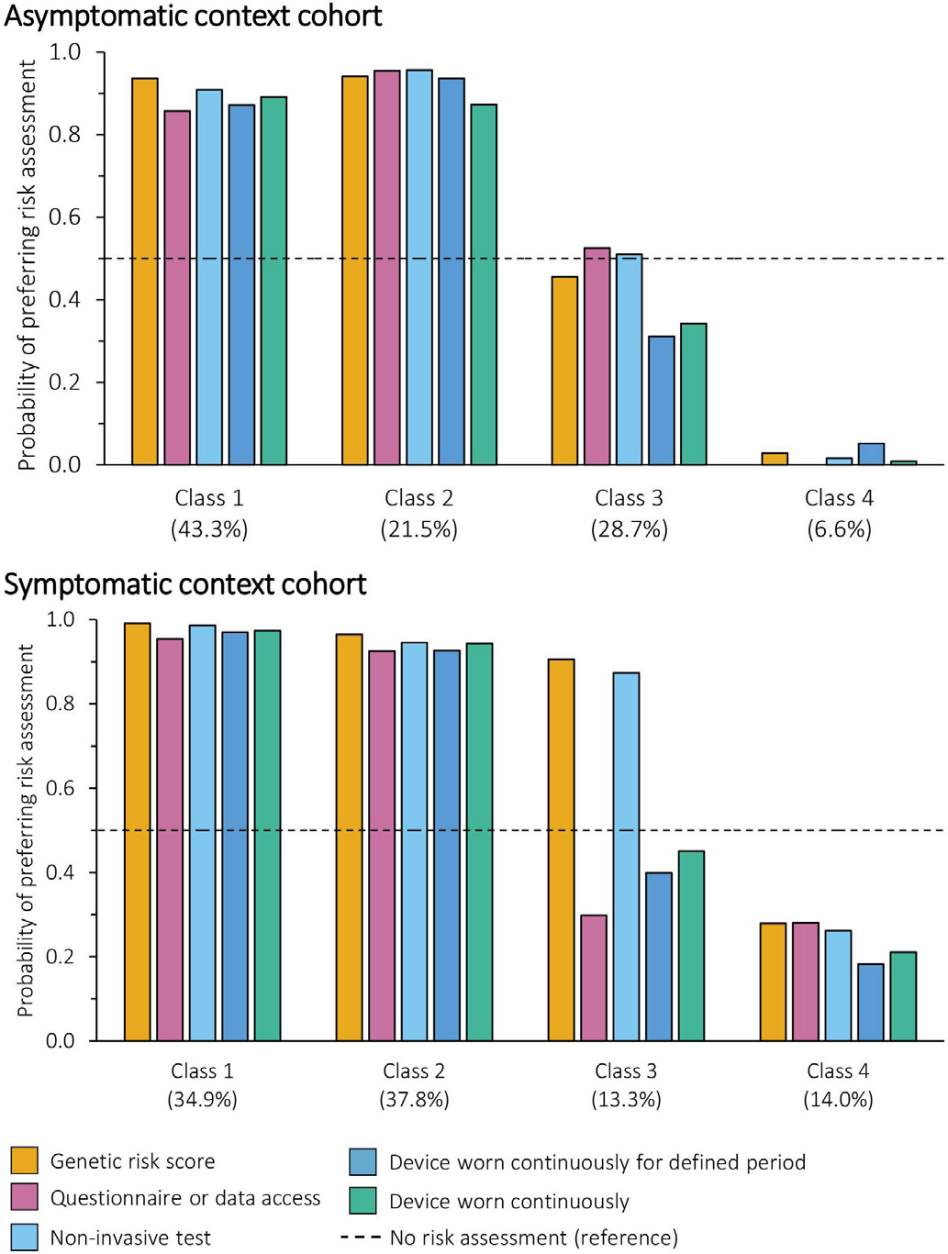
Probability of preferring different risk assessments with a fixed accuracy by class membership (latent class analysis). Each example of risk-based innovation and the reference was modelled as (method, type, location, frequency, sensitivity and specificity): (1) genetic risk score: blood test, genetic, general practice, one-off single event, 87.5%. (2) Questionnaire or data access: questionnaire or data access, non-genetic, home, once every 5 years, 87.5%. (3) Non-invasive test (saliva or blood test): non-invasive test, non-genetic, community, once every year, 87.5%. (4) Device worn continuously for defined period: wearable device, non-genetic, hospital, continuously for a 2 week period, 87.5%. (5) Device worn continuously: wearable device, non-genetic, community, constantly, 87.5%. (6) No risk assessment (reference): none, none, none, none, 87.5% (plus opt-out coefficient).

Four latent classes were identified in both the asymptomatic and symptomatic context cohorts. For the asymptomatic context cohort, sex (male or female) and personal history of cancer (history or no history) were included in the final model; for the symptomatic context cohort, sex (male or female) was included in the final model. The full results are reported in online supplemental table 7. Figure 5 illustrates the probability of each class taking up the different risk assessments at a fixed sensitivity and specificity of 87.5%.

In the asymptomatic context, the majority of participants fell into Classes 1 and 2 and favoured risk assessments, with a probability of choosing any modelled risk assessment of at least 85%. Class 1 (43.3% of the cohort) was characterised by preferences for tests or wearable devices that are completed at home. Conversely, Class 2 (21.1% of the cohort) was characterised by preferences for questionnaire or data access-based methods that are not completed at home, and sensitivity was much more important to them than specificity. A quarter of participants were in Class 3 and expressed no preference for or against risk assessments (coefficient of the constant −0.150, p=0.432). Instead, sensitivity and specificity, not attending the hospital and not using wearable devices were important. This means they were unlikely to take up the risk assessments at the levels modelled, particularly when a device would need to be worn. Finally, 6.6% of the cohort (Class 4) were strongly averse to risk assessments, regardless of the attributes. Although this was identified as the best model, sex and history of cancer did not predict class membership, with the exception of participants in Class 1 being less likely to be female than Class 4.

In the symptomatic context cohort, participants in Classes 1 and 2 (34.9% and 37.8% of the cohort, respectively) would select a risk assessment 90% of the time, regardless of the innovation. The choice of Class 1 was driven by maximising accuracy, whereas participants in Class 2 considered multiple attributes, particularly the method and whether the test was genetic, alongside sensitivity and specificity. Conversely, the two smallest classes were composed of people who preferred no risk assessments: when they did choose a risk assessment, participants in Class 4 (14.0% of the cohort) prioritised sensitivity and specificity, whereas participants in Class 3 (13.3% of the cohort) favoured blood and non-invasive tests, which drove support for genetic and non-invasive testing and made these the only tests they were likely to take up. Participants who preferred no risk assessment (Classes 3 and 4) were more likely to be female sex than Classes 1 and 2.

#### Self-reported acceptability and preferences for risk assessments

Participants’ expressed views on the acceptability of using cancer risk assessments reflected the analysis from the main DCE (online supplemental table 8). The majority (59%, n=708 participants) felt that cancer risk assessments in both contexts were acceptable. Of the remaining participants, more than twice as many felt that it was more acceptable in the symptomatic than asymptomatic context (26%, n=317 participants vs 11%, n=135 participants). A minority (3.5%, n=42 participants) considered risk assessments to be acceptable in neither context. There were no differences between the views according to the context in which they completed the DCE (p=0.364).

Finally, online supplemental figure 3 shows the order in which participants ranked the attributes of risk assessments. This largely follows the relative importance of the attributes revealed in the DCE, although these rankings do not account for the levels of the attributes.

## Discussion

We have demonstrated support for risk assessments using novel technologies and innovations to inform investigations of vague symptoms and also within cancer screening programmes. We found that a high proportion of the public would be extremely likely to take up risk assessments and stratified healthcare regardless of the assessment used. Some people would only take up risk assessments under certain circumstances, depending on test methods and performance metrics, and a minority were against them. Overall, this provides evidence to support policymakers in taking steps towards implementing such tests within screening and primary care.

This DCE adds to the growing literature on the public’s attitudes towards risk-stratified healthcare within cancer early diagnosis and screening by focusing on the use of innovations to estimate personal cancer risk. Our findings of public prioritisation of sensitivity, and secondarily method of testing and specificity, are consistent with previous studies.^29^,^31^ More surprising was the unimportance of location and test frequency, suggesting that the public are willing to be inconvenienced as long as sensitivity and specificity are at a minimum 80% and blood and non-invasive tests (or wearable devices in the symptomatic context) are used. Our qualitative research suggests that while a lower burden of testing is favoured, concepts like perceived effectiveness and invention coherence are more important.^24^ This suggests that these priorities can be reflected by those developing and implementing these technologies.

Our modelled preferences for a range of risk-based innovations using the DCE results reflect some of the diversity of innovations in this field.^5^ Across all features (not just test method), preference for genetic and non-genetic tests via blood or saliva samples aligns with observed public willingness to participate in genetic testing^18 19^ and positivity towards multi-cancer early detection blood tests that are a comparatively less-invasive screening strategy.^32^ Such tests may be considered logical since providing samples is a normal part of screening and medical investigations. More qualitative research is needed to illuminate the reasons for the relative popularity of 5 yearly questionnaires and analysis of existing medical records by context; a balance between burden and perceived reliability, and existing knowledge and beliefs about the accuracy of the test methods is likely to come into play. Finally, the less-favourable perspectives towards continually worn wearable devices in the asymptomatic context align with ongoing research regarding whether they will substantially contribute to health promotion through feedback and monitoring and/or create a ‘cyborg, post-human self’ with biometrics overriding individual perspectives.^33^ Nevertheless, they could be viewed as a way of monitoring symptoms in those with concerns.

Another key finding is the high proportion of the public (Classes 1 and 2: 65% in the asymptomatic context and 73% in the symptomatic context) that would be extremely likely to participate in risk assessments and stratified healthcare regardless of the assessment used, plus others who would only take up certain offers. Despite different priorities for certain levels within each context, the variability in overall likelihood of taking up the different examples of risk assessments was small (maximum 10% difference) at a fixed sensitivity and specificity. Therefore, it is, perhaps, for Class 3 that product development, user testing, information about the test and sensitivity and specificity will be most important as their participation would depend on the risk assessment in question.

There is also a subset who are unwilling to complete risk assessments for this purpose (Class 4): 7% of participants for the asymptomatic context and 14% for symptomatic (although the strength of support for the no risk assessment option was much greater for asymptomatic than symptomatic). For these groups, optimising risk assessment formats or performance is unlikely to encourage attendance. Other avenues will therefore need to be developed to engage this group, as well as a clear protocol for those who decline risk assessment. With the exception of sex, we were not able to describe this group by easily identified characteristics. Therefore, further research is also needed to understand whether unifying features exist and to understand the reasons behind this perspective. Given somewhat unsympathetic public perspectives towards those who do not take up screening or complete risk assessments—that such individuals are irresponsible and should be deprioritised for screening—this will be important.^34 35^

Regarding the strengths and limitations for this study, we collected the views of a relatively large sample that was representative of the UK population with regard to age, sex, ethnicity and self-reported socioeconomic status. Nevertheless, it is likely that receptiveness to risk-based innovations has been somewhat overestimated because people who are able to (eg, due to computer access) and want to take part in research on this topic may be more likely to engage with healthcare than those who do not. Splitting the sample into asymptomatic and symptomatic contexts enabled us to compare views towards different uses of risk assessments. We did not specify the examples of innovations by name but classified them using a range of attributes. Although this has advantages such as making the findings relevant to a wider range of novel technologies (specifically, imputing acceptability for other technologies using the coefficients generated in the DCE), it means we have not accounted for preconceptions such as perspectives towards artificial intelligence.^36^ Furthermore, there are many other relevant attributes of innovations that we did not include to make it a manageable exercise for participants nor give participants the option to opt out of screening altogether. We also did not separate completion of risk assessments from risk-stratified healthcare as this is most realistic in the context of universal healthcare systems. Lastly, we observed that participants spent little time reading the instructions and background information (median 15 s, IQR 9 to 23 s); therefore, they did not take the opportunity to learn about the concepts included in the DCE. Nonetheless, three-quarters of participants answered all three comprehension questions correctly, and they took a median of 15 min to complete the whole survey.

### Conclusion

We have shown a high level of public support for risk-based innovations within risk-stratified healthcare for early diagnosis, particularly in the context of referral decision-making. Innovations that use blood tests and non-invasive samples are most likely to be preferred across the population. With sensitivity and specificity at 87.5%, over 86% of the population were likely to take them up if they have symptoms and 65% are likely to take them up to inform screening (which could increase to 94% with optimal sensitivity and specificity). A minority showed strong dislike of risk-based innovations, particularly within screening. Therefore, work is needed to understand who these people are and how to involve them. Optimising and communicating such risk-based innovations should be prioritised to engage those whose participation is dependent on test methods and performance metrics.

## Supplementary material

10.1136/bmjopen-2024-093803 online supplemental file 1

## Data Availability

Data are available in a public, open access repository.
