# Supplementary material for: Risk-based innovations in cancer screening and diagnosis: a discrete choice experiment to explore priorities of the UK public
Source: BMJ Open. 2025 May 31;15(5):e093803. doi: 10.1136/bmjopen-2024-093803 (PMC12142028; doi:10.1136/bmjopen-2024-093803)
Supplement: online supplemental file 1 [file bmjopen-15-5-s001.pdf]

Priorities of the UK public for risk-based innovations in cancer screening and referral to investigate symptoms: a discrete choice experiment

Rebecca A. Dennison, Reanna J. Clune, Stephen Morris, Jo Waller, Juliet A. Usher-Smith

Supplementary material

1. **Supplementary Table 1.** Full question presented in the DCE.

2. **Supplementary Table 2.** DCE question matrix.

3. **Supplementary Table 3.** Details of model selection.

4. **Supplementary Figure 1.** Impact of varying the nonsense sensitivity and specificity variables for no risk assessments on predicted probabilities.

5. **Supplementary Table 4.** Number of correct responses to the comprehension questions.

6. **Supplementary Table 5.** Participants' thoughts and beliefs about cancer and screening.

7. **Supplementary Figure 2.** Participants' ease of completing the DCE.

8. **Supplementary Table 6.** Participants' preferences for risk assessments in screening and referral context cohorts (sensitivity analyses).

9. **Supplementary Table 7.** Participants' preferences for risk assessments in screening and referral context cohorts by class (latent class analysis).

10. **Supplementary Table 8.** Participants' reported views on the relative acceptability of using cancer risk assessments in screening and referral contexts.

11. **Supplementary Figure 3.** Participants' ranking of the attributes of risk assessments.

1. Supplementary Table 1. Full question presented in the DCE.

Example choice set:

	Option 1	Option 2
**Method of risk assessment**	**Wearable device**<br>A device like a smartwatch, patch or sensor is worn to continuously monitor factors such as sleep patterns, heart rate and temperature or to monitor biomarker levels (which are signals of what is going on in the body).	**Questionnaire or data access**<br>Cancer risk can be estimated using data from the person's existing health records (with their permission), or they could do a questionnaire to provide additional information.
**Type of risk assessment**	**Non-genetic**<br>Data other than a person's genes or DNA are analysed to estimate their cancer risk.	**Non-genetic**<br>Data other than a person's genes or DNA are analysed to estimate their cancer risk.
**Location of risk assessment**	**Community clinic/pharmacy**<br>The person would go to a community clinic (in a supermarket, for example) or pharmacy to have the wearable device set up.	**Home**<br>The person would do the questionnaire/give permission to access data in their own home.
**Frequency of risk assessment**	**Continuously for a 2-week period**<br>They would wear the device continuously over a two-week period.	**Constantly**<br>They would give permission for data access continuously over a prolonged period of time and update the questionnaire if anything changed.
**Accuracy – risk of cancer is over-estimated**	**10 out of every 100 people** who have a risk assessment are **told they are at high risk of cancer when actually they are not.**	**5 out of every 100 people** who have a risk assessment are **told they are at high risk of cancer when actually they are not.**
	This means that they might be offered more screening than they should be, based on their actual cancer risk. The tests may cause more harm than benefit.	
**Accuracy – risk of cancer is under-estimated**	**20 out of every 100 people** who have a risk assessment are **told they are at low risk of cancer when actually their risk is higher.**	**10 out of every 100 people** who have a risk assessment are **told they are at low risk of cancer when actually their risk is higher.**
	This means they might be offered less screening than they should be, based on their actual cancer risk. This might mean a cancer is diagnosed later than it could have been if they had been offered more tests.	

Full question in the **asymptomatic context**:

Imagine someone has no symptoms of cancer. A decision must be made about the age at which they are first invited to screen for a particular type of cancer, and how often they should be invited.

Which option do you think is most acceptable?

1. Using their risk estimated according to **Option 1**, offer more intensive screening if they have a high risk and less intensive screening if they have a low risk.

2. Using their risk estimated according to **Option 2**, offer more intensive screening if they have a high risk and less intensive screening if they have a low risk.

3. **Neither** – do not estimate their risk and so offer the same screening to everyone (at average intensity).

Full question in the **symptomatic context**:

Imagine someone has a symptom that could potentially be a cancer. A decision needs to be made about which referral to make to investigate their symptoms. What investigations they should be offered must be decided.

Which option do you think is most acceptable?

1. Using their risk estimated according to **Option 1 alongside the clinical judgement of the GP**, arrange urgent, extensive tests if they have a high risk and refer initially for less urgent, less extensive tests if they have a low risk.

2. Using their risk estimated according to **Option 2 alongside the clinical judgement of the GP**, arrange urgent, extensive tests if they have a high risk and initially refer for a non-urgent test if they have a low risk.

3. **Neither** – do not estimate their risk and refer based on clinical judgment alone.

2. Supplementary Table 2. DCE question matrix.

Block	Choice set	Alternative	Method	Type	Location	Frequency	Overestimated risk*	Underestimated risk*
1	1	1	Data	Non-genetic	Community	Constantly	15	15
		2	Data	Non-genetic	Home	Once every year	20	20
2	2	1	Wearable device	Non-genetic	Home	Constantly	20	5
		2	Non-invasive test	Non-genetic	Hospital	Once every year	5	20
2	3	1	Non-invasive test	Genetic	Home	One-off single event	10	5
		2	Data	Non-genetic	General practice	One-off single event	20	15
2	4	1	Blood test	Genetic	Hospital	One-off single event	20	20
		2	Wearable device	Non-genetic	Home	Continuously for 2 weeks	5	15
1	5	1	Non-invasive test	Non-genetic	General practice	Once every 5 years	10	10
		2	Blood test	Non-genetic	Community	Once every year	15	5
2	6	1	Non-invasive test	Genetic	General practice	One-off single event	15	15
		2	Blood test	Non-genetic	Community	Once every 5 years	10	10
1	7	1	Wearable device	Non-genetic	Hospital	Continuously for 2 weeks	20	10
		2	Data	Non-genetic	Home	Once every year	10	15
1	8	1	Blood test	Genetic	General practice	One-off single event	15	20
		2	Non-invasive test	Non-genetic	Hospital	One-off single event	20	5
2	9	1	Data	Non-genetic	Hospital	One-off single event	10	10
		2	Non-invasive test	Non-genetic	Community	Once every 5 years	20	5
1	10	1	Wearable device	Non-genetic	Community	Constantly	5	20
		2	Blood test	Non-genetic	General practice	Once every 5 years	15	5
1	11	1	Wearable device	Non-genetic	General practice	Constantly	15	20
		2	Blood test	Genetic	Community	One-off single event	20	15
1	12	1	Wearable device	Non-genetic	Community	Continuously for 2 weeks	10	20
		2	Data	Non-genetic	Home	Constantly	5	10
1	13	1	Data	Non-genetic	General practice	Once every year	5	5
		2	Non-invasive test	Non-genetic	Home	One-off single event	15	20
2	14	1	Data	Non-genetic	Community	Once every year	15	10
		2	Blood test	Non-genetic	General practice	One-off single event	5	5
2	15	1	Non-invasive test	Non-genetic	Community	One-off single event	5	10
		2	Data	Non-genetic	Hospital	Constantly	10	15
2	16	1	Wearable device	Non-genetic	Hospital	Constantly	15	15
		2	Data	Non-genetic	Home	Constantly	20	20
1	17	1	Data	Non-genetic	Community	One-off single event	10	5
		2	Blood test	Non-genetic	Hospital	Once every year	20	15
2	18	1	Non-invasive test	Non-genetic	General practice	Once every year	10	10
		2	Data	Non-genetic	Hospital	Once every 5 years	5	20

** Accuracy – people out of 100 whose risk will be over- or underestimated.*

3. Supplementary Table 3. Details of model selection.

	Screening context (n=601)			Referral context (n=601)		
	Log likelihood	AIC	BIC	Log likelihood	AIC	BIC
a. Model						
Basic conditional logistic model	-5363.226	10754.45	10862.17	-4683.629	9395.26	9502.98
Including constants for options 1 and 2	-5363.046	10756.09	10871.51	-4682.038	9394.08	9509.49
Dummy coded overestimated risk	-5362.497	10756.99	10880.1	-4681.938	9395.88	9518.99
Dummy coded underestimated risk	-5362.086	10756.17	10879.28	-4677.620	9387.24	9510.35
b. Number of classes for latent class analysis						
2 classes	-4879.089	9816.178	10039.32	-4266.114	8590.228	8813.367
3 classes	-4742.984	9573.969	9912.524	-4147.905	8383.810	8722.365
4 classes	-4661.878	9441.756	9895.728	-4051.602	8221.205	8675.176
5 classes	-4606.049	9360.098	9929.486	*Did not converge*		
c. Seed						
Default	-4661.878	9441.756	9895.728	-4051.602	8221.205	8675.176
39	-4651.219	9420.438	9874.410	*Did not converge*		
45	-4661.876	9441.752	9895.724	-4051.603	8221.205	8675.177
65	-4651.220	9420.439	9874.411	-4051.602	8221.205	8675.176
67	-4661.878	9441.756	9895.728	-4051.603	8221.206	8675.177
200	-4651.219	9420.438	9874.410	-4055.906	8229.811	8683.783
1234	-4661.878	9441.756	9895.728	-4105.510	8329.021	8782.992
5679	-4661.876	9441.751	9895.723	*Did not converge*		
d. Class membership*						
Original model	-4651.220	9420.439	9679.956	-4051.602	8221.205	8480.722
Over 50 years	-4644.118	9412.236	9684.949	-4046.534	8217.067	8489.780
Female sex	-4641.557	9407.115	9679.828	-4041.566	8207.133	8479.846
Ethnicity white	-4648.129	9420.258	9692.971	-4048.302	8220.604	8493.317
Low self-reported socioeconomic status	-4651.104	9426.209	9698.921	-4055.916	8235.833	8508.545
Degree education	-4649.532	9423.063	9695.776	-4052.023	8228.046	8500.759
Never smoked	-4646.829	9417.658	9690.371	-4048.196	8220.393	8493.106
Overweight	-4650.132	9424.264	9696.977	-4103.594	8331.187	8603.900
Cancer history	-4618.638	9361.275	9633.988	-4044.159	8212.317	8485.030
Attended screening	-4648.814	9421.627	9694.340	-4046.150	8216.301	8489.014
Worried about cancer	-4649.570	9423.140	9695.853	-4054.265	8232.351	8505.243
Think likely to get cancer	-4650.072	9424.143	9696.856	-4057.089	8238.177	8510.890

AIC: Akaike Information Criterion; BIC: Bayesian Information Criterion.
**601 used in calculating BIC.*
Models with the lowest values of the AIC and BIC are highlighted in green.

4. Supplementary Figure 1. Impact of varying the nonsense sensitivity and specificity variables for no risk assessments on predicted probabilities.

Asymptomatic context cohort

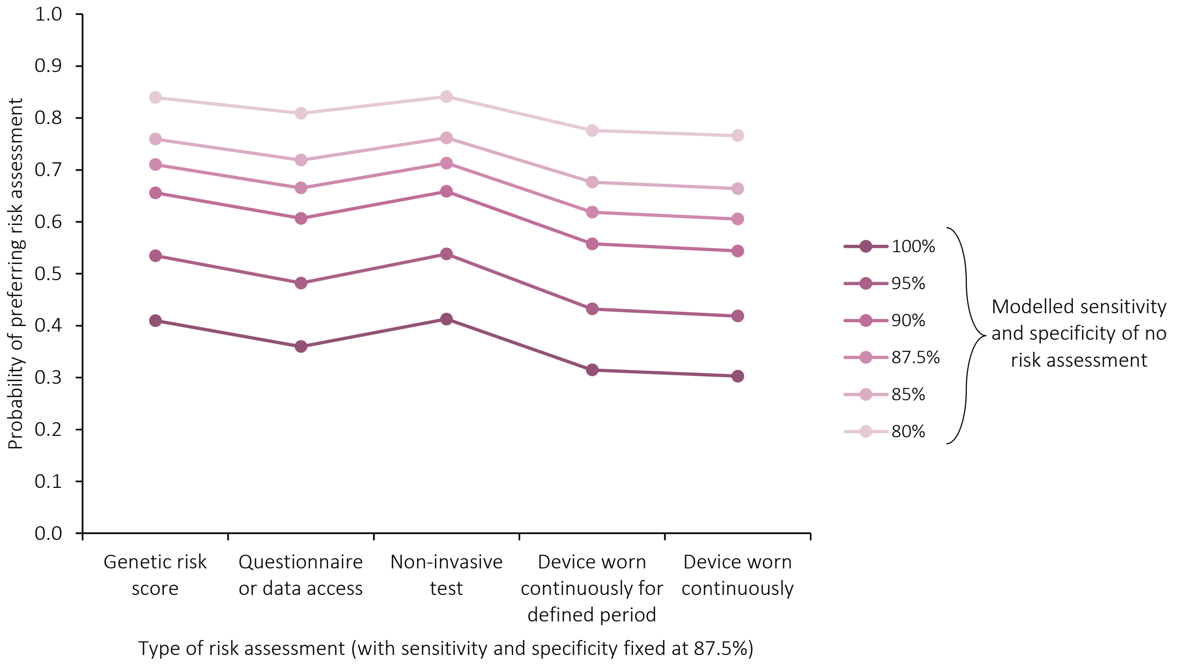

Symptomatic context cohort

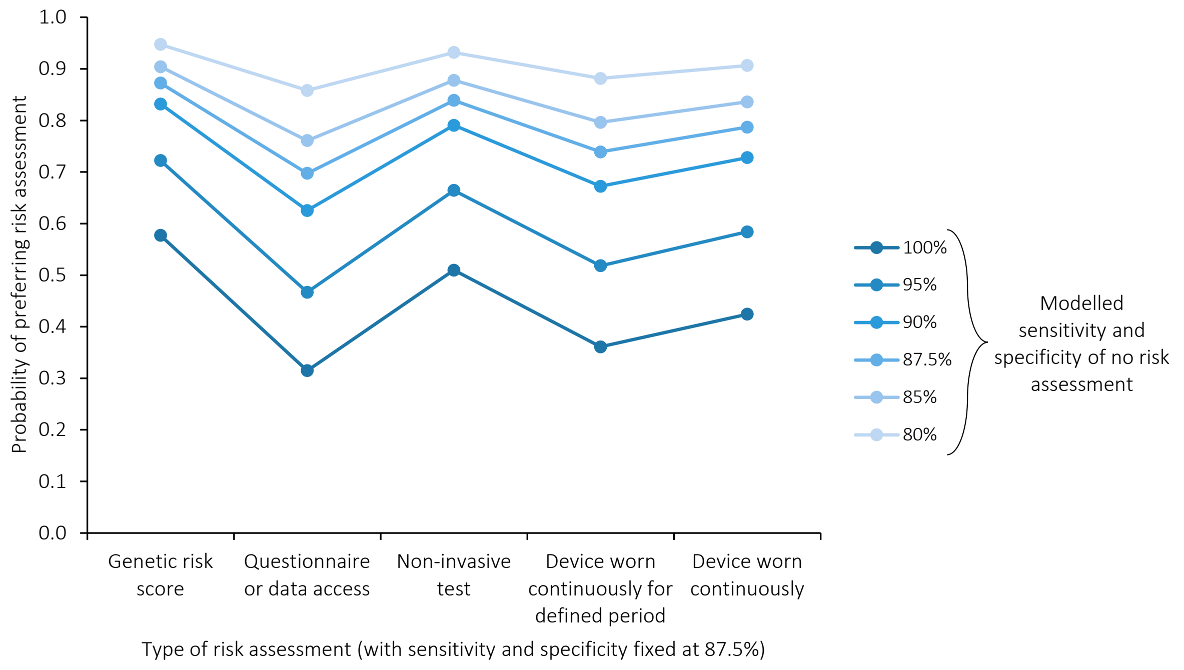

5. Supplementary Table 4. Number of correct responses to the comprehension questions.

	Asymptomatic context cohort	Symptomatic context cohort	Total (%)
Total *N*	601	601	1,202 (100.0)
Q1. People may need to give a saliva or stool sample for Option 1 [true]			
Correct	594	596	1,190 (99.0)
Incorrect	7	5	12 (1.0)
Q2. More people would have their risk of cancer over-estimated and would be screened/tested more intensely than necessary in Option 2 compared to Option 1 [true]			
Correct	476	468	944 (78.5)
Incorrect	125	133	258 (21.5)
Q3. People would have to repeat the test in Option 1 quite often [false]			
Correct	552	36	1,117 (92.9)
Incorrect	49	565	85 (7.1)
Total number of questions answered correctly			
Zero or one	15	20	35 (2.9)
Two	150	134	284 (23.6)
Three	436	447	883 (73.5)

6. Supplementary Table 5. Participants' thoughts and beliefs about cancer and screening.

	Asymptomatic context cohort	Symptomatic context cohort	Total (%)
Total *N*	601	601	1,202 (100.0)
"These days, many people with cancer can expect to continue with normal activities and responsibilities"			
Strongly agree	48	36	84 (7.0)
Agree	324	335	659 (54.8)
Neither agree nor disagree	159	151	310 (25.8)
Disagree	65	73	138 (11.5)
Strongly disagree	5	6	11 (0.9)
"Most cancer treatment is worse than the cancer itself"			
Strongly agree	27	30	57 (4.8)
Agree	139	125	264 (22.0)
Neither agree nor disagree	205	214	419 (34.9)
Disagree	187	181	368 (30.6)
Strongly disagree	43	51	94 (7.8)
"I would not want to know if I had cancer"			
Strongly agree	14	12	26 (2.2)
Agree	25	22	47 (3.9)
Neither agree nor disagree	63	65	128 (10.7)
Disagree	197	181	378 (31.5)
Strongly disagree	302	321	623 (51.8)
"Cancer can often be cured"			
Strongly agree	79	79	158 (13.1)
Agree	303	312	615 (51.2)
Neither agree nor disagree	171	146	317 (26.4)
Disagree	41	58	99 (8.2)
Strongly disagree	7	6	13 (1.1)
"Going to the doctor as quickly as possible after noticing a symptom of cancer could increase the chances of surviving"			
Strongly agree	412	432	844 (70.2)
Agree	170	144	314 (26.1)
Neither agree nor disagree	9	18	27 (2.3)
Disagree	4	5	9 (0.8)
Strongly disagree	6	2	8 (0.7)
"Some people think that a diagnosis of cancer is a death sentence"			
Strongly agree	28	28	56 (4.7)
Agree	137	129	266 (22.1)
Neither agree nor disagree	189	165	354 (29.5)
Disagree	212	223	435 (36.2)
Strongly disagree	35	56	91 (7.6)
How likely do you think is it that you will get cancer at some point in the next 10 years?			
Extremely or moderately likely	114	135	249 (20.7)
Slightly likely	153	157	310 (25.8)
Neither likely nor unlikely	195	148	343 (28.5)
Slightly unlikely	41	57	98 (8.2)
Extremely or moderately unlikely	98	104	202 (16.8)
During the past month, how often have you thought about your own chances of getting cancer?			
Not at all	192	194	386 (32.1)
Rarely	191	177	368 (30.6)
Sometimes	158	159	317 (26.4)
Often or a lot	60	71	131 (10.9)

During the past month, how often have thoughts about your chances of getting cancer affected your mood?			
Not at all	325	339	664 (55.2)
Rarely	159	140	299 (24.9)
Sometimes	83	92	175 (14.5)
Often or a lot	34	30	64 (5.3)
During the past month, how often have thoughts about your chances of getting cancer affected your ability to perform your daily activities?			
Not at all	437	456	893 (74.3)
Rarely	104	95	199 (16.6)
Sometimes	39	37	76 (6.3)
Often or a lot	21	13	34 (2.8)

7. Supplementary Figure 2. Participants' ease of completing the DCE.

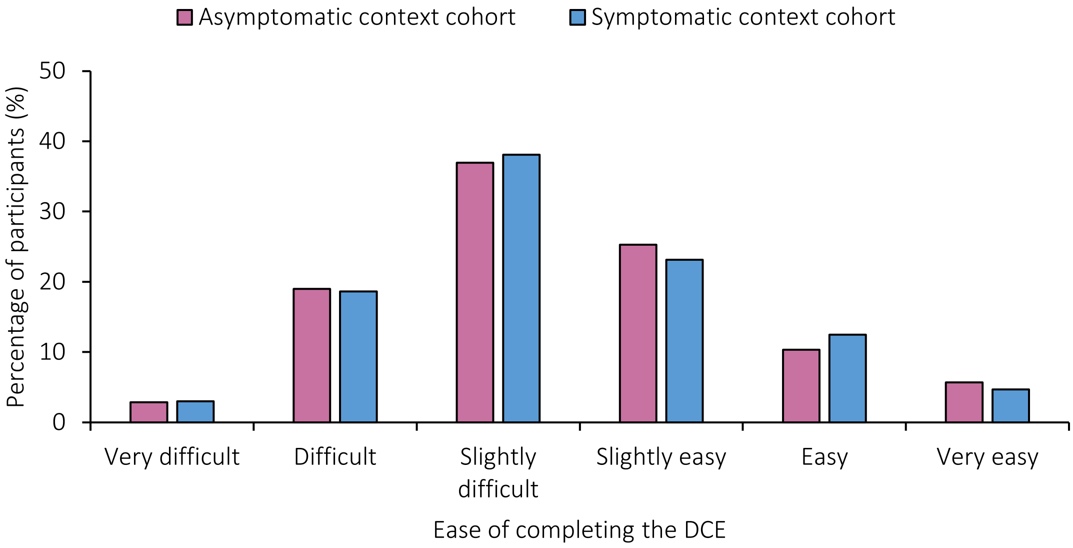

8. Supplementary Table 6. Participants' preferences for risk assessments in screening and referral context cohorts (sensitivity analyses).

	a. Participants who paid attention by completing the survey in at least 7.5 minutes and not always selecting Option 1 or Option 2			b. Participants who showed understanding of the concepts by answering all of the comprehension questions correctly		
	Asymptomatic context cohort	Symptomatic context cohort	p value for difference between cohorts	Asymptomatic context cohort	Symptomatic context cohort	p value for difference between cohorts
N participants	565	560	**<0.001** for	436	447	**<0.001** for
N observations	15,255	15,120	overall	11,772	12,069	overall
Pseudo R^2	0.1045	0.2205	difference	0.1057	0.2256	difference
Constant (no risk assessment)	**-0.677 (-0.825 to -0.529)**	**-0.802 (-0.965 to -0.640)**	0.264	**-0.611 (-0.779 to -0.442)**	**-0.762 (-0.946 to -0.577)**	0.236
**Method of risk assessment**						
Questionnaire or data access	Reference	Reference		Reference	Reference	
Blood test	**0.336 (0.206 to 0.466)**	**1.024 (0.881 to 1.167)**	**<0.001**	**0.348 (0.200 to 0.495)**	**0.987 (0.825 to 1.149)**	**<0.001**
Non-invasive test	**0.339 (0.226 to 0.452)**	**0.765 (0.645 to 0.885)**	**<0.001**	**0.401 (0.272 to 0.529)**	**0.701 (0.566 to 0.837)**	**0.002**
Wearable device	-0.164 (-0.339 to 0.012)	**0.258 (0.084 to 0.433)**	**0.001**	-0.143 (-0.343 to 0.056)	**0.281 (0.088 to 0.474)**	**0.003**
**Type of risk assessment**						
Non-genetic	Reference	Reference		Reference	Reference	
Genetic	0.021 (-0.132 to 0.174)	0.120 (-0.048 to 0.289)	0.392	0.009 (-0.165 to 0.183)	**0.198 (0.008 to 0.388)**	0.150
**Location of risk assessment**						
Home	Reference	Reference		Reference	Reference	
Community clinic/pharmacy	-0.047 (-0.164 to 0.070)	0.095 (-0.029 to 0.219)	0.103	0.060 (-0.073 to 0.192)	0.120 (-0.020 to 0.260)	0.539
General practice	-0.106 (-0.228 to 0.015)	-0.027 (-0.158 to 0.105)	0.383	-0.018 (-0.156 to 0.119)	0.018 (-0.131 to 0.168)	0.724
Hospital	**-0.231 (-0.345 to -0.117)**	-0.039 (-0.151 to 0.072)	0.019	**-0.184 (-0.314 to -0.054)**	-0.041 (-0.167 to 0.085)	0.122
**Frequency of risk assessment**						
One-off single event	Reference	Reference		Reference	Reference	
Once every 5 years	0.001 (-0.138 to 0.140)	0.001 (-0.151 to 0.153)	0.998	0.010 (-0.149 to 0.168)	0.035 (-0.135 to 0.204)	0.834
Once every year	-0.042 (-0.162 to 0.077)	-0.007 (-0.138 to 0.124)	0.698	-0.058 (-0.195 to 0.078)	0.039 (-0.111 to 0.188)	0.349
Continuously for 2 weeks	0.179 (-0.079 to 0.438)	-0.016 (-0.275 to 0.243)	0.295	0.158 (-0.136 to 0.452)	0.077 (-0.211 to 0.364)	0.699
Constantly	-0.042 (-0.211 to 0.128)	0.109 (-0.069 to 0.287)	0.230	0.017 (-0.175 to 0.209)	0.180 (-0.019 to 0.378)	0.249
**Accuracy**						
Specificity	**0.045 (0.038 to 0.053)**	**0.051 (0.043 to 0.059)**	**0.273**	**0.045 (0.037 to 0.054)**	**0.053 (0.044 to 0.062)**	0.207
Sensitivity	**0.064 (0.056 to 0.071)**	**0.086 (0.078 to 0.093)**	**<0.001**	**0.061 (0.053 to 0.069)**	**0.090 (0.081 to 0.098)**	**<0.001**
**p value for difference versus main analysis**						
	**0.003**	**<0.001**		0.153	**0.025**	

9. Supplementary Table 7. Participants' preferences for risk assessments in screening and referral context cohorts by class (latent class analysis).

Asymptomatic context cohort*

	Class 1 (43.3%)		Class 2 (21.5%)		Class 3 (28.7%)		Class 4 (6.6%)	
	Coefficient	P value	Coefficient	P value	Coefficient	P value	Coefficient	P value
Constant (no risk assessment)	**-1.831 (-2.305 to -1.356)**	**<0.001**	**-2.802 (-3.552 to -2.052)**	**<0.001**	-0.150 (-0.523 to 0.224)	0.432	**6.820 (2.304 to 11.335)**	**0.003**
Method of risk assessment								
Questionnaire or data access	Reference		Reference		Reference		Reference	
Blood test	**1.313 (0.961 to 1.665)**	**<0.001**	**-1.010 (-1.508 to -0.512)**	**<0.001**	-0.113 (-0.513 to 0.286)	0.578	-1.640 (-4.824 to 1.543)	0.312
Non-invasive test	**1.045 (0.789 to 1.302)**	**<0.001**	**-0.672 (-1.162 to -0.182)**	**0.007**	0.074 (-0.227 to 0.375)	0.629	-2.143 (-5.086 to 0.800)	0.154
Wearable device	**1.135 (0.753 to 1.518)**	**<0.001**	**-2.378 (-3.217 to -1.540)**	**<0.001**	**-0.524 (-1.017 to -0.031)**	**0.037**	-1.323 (-4.344 to 1.698)	0.391
Type of risk assessment								
Non-genetic	Reference		Reference		Reference		Reference	
Genetic	0.026 (-0.282 to 0.334)	0.871	0.226 (-0.231 to 0.683)	0.333	-0.180 (-0.533 to 0.174)	0.319	3.973 (-0.996 to 8.942)	0.117
Location of risk assessment								
Home	Reference		Reference		Reference		Reference	
Community clinic/pharmacy	**-0.440 (-0.688 to -0.191)**	**0.001**	**0.815 (0.322 to 1.309)**	**0.001**	-0.039 (-0.325 to 0.247)	0.791	2.527 (-0.186 to 5.239)	0.068
General practice	**-0.475 (-0.730 to -0.220)**	**<0.001**	**0.759 (0.265 to 1.254)**	**0.003**	-0.034 (-0.324 to 0.256)	0.819	0.946 (-2.008 to 3.901)	0.530
Hospital	**-0.707 (-0.933 to -0.480)**	**<0.001**	**0.826 (0.390 to 1.262)**	**<0.001**	**-0.334 (-0.647 to -0.021)**	**0.036**	2.798 (-0.263 to 5.859)	0.073
Frequency of risk assessment								
One-off single event	Reference		Reference		Reference		Reference	
Once every 5 years	-0.037 (-0.306 to 0.232)	0.786	0.252 (-0.180 to 0.683)	0.253	-0.047 (-0.355 to 0.261)	0.764	-41.145 (-88.169 to 5.878)	0.086
Once every year	-0.141 (-0.370 to 0.088)	0.227	0.147 (-0.287 to 0.580)	0.507	-0.146 (-0.411 to 0.118)	0.278	2.326 (-1.102 to 5.755)	0.184
Continuously for 2 weeks	-0.346 (-0.828 to 0.137)	0.160	**1.442 (0.584 to 2.299)**	**0.001**	-0.085 (-0.701 to 0.532)	0.788	2.441 (-2.307 to 7.189)	0.314
Constantly	**-0.424 (-0.768 to -0.081)**	**0.016**	0.688 (-0.017 to 1.394)	0.056	-0.239 (-0.626 to 0.147)	0.225	0.812 (-2.529 to 4.154)	0.634
Accuracy								
Specificity	**0.066 (0.051 to 0.082)**	**<0.001**	**0.029 (0.005 to 0.053)**	**0.017**	**0.056 (0.038 to 0.074)**	**<0.001**	0.153 (-0.043 to 0.350)	0.125
Sensitivity	**0.045 (0.032 to 0.058)**	**<0.001**	**0.105 (0.069 to 0.140)**	**<0.001**	**0.091 (0.069 to 0.112)**	**<0.001**	0.059 (-0.250 to 0.133)	0.548
Class membership								
Female sex (versus male)	**-0.842 (-1.574 to -0.111)**	**0.024**	-0.433 (-1.225 to 0.358)	0.283	-0.491 (-1.251 to 0.269)	0.206	Reference	
History of cancer (versus no history of cancer)	0.594 (-0.919 to 2.107)	0.442	-14.028 (-384.506 to 356.450)	0.941	0.366 (-1.197 to 1.929)	0.646	Reference	

**Latent class analysis limited to 40 iterations.*

Symptomatic context cohort

	Class 1 (34.9%)		Class 2 (37.8%)		Class 3 (13.3%)		Class 4 (14.0%)	
	Coefficient	P value	Coefficient	P value	Coefficient	P value	Coefficient	P value
Constant (no risk assessment)	-2.974 (-3.748 to -2.201)	<0.001	-2.382 (-2.806 to -1.957)	<0.001	1.283 (0.012 to 2.554)	0.048	0.910 (0.317 to 1.503)	0.003
**Method of risk assessment**								
Questionnaire or data access	Reference		Reference		Reference		Reference	
Blood test	**1.446 (0.755 to 2.136)**	**<0.001**	**0.457 (0.155 to 0.759)**	**0.003**	**3.047 (2.040 to 4.054)**	**<0.001**	-0.015 (-0.592 to 0.562)	0.960
Non-invasive test	**1.066 (0.504 to 1.628)**	**<0.001**	**0.373 (0.131 to 0.614)**	**0.002**	**2.642 (1.482 to 3.802)**	**<0.001**	-0.102 (-0.580 to 0.376)	0.676
Wearable device	0.181 (-0.391 to 0.753)	0.535	**0.312 (0.004 to 0.621)**	**0.047**	0.819 (-0.310 to 1.949)	0.155	-0.653 (-1.357 to 0.052)	0.069
**Type of risk assessment**								
Non-genetic	Reference		Reference		Reference		Reference	
Genetic	0.181 (-0.638 to 0.999)	0.665	**0.371 (0.122 to 0.619)**	**0.003**	0.816 (-0.100 to 1.732)	0.081	-0.016 (-0.625 to 0.593)	0.960
**Location of risk assessment**								
Home	Reference		Reference		Reference		Reference	
Community clinic/pharmacy	0.248 (-0.255 to 0.751)	0.334	0.082 (-0.117 to 0.280)	0.420	0.105 (-0.542 to 0.751)	0.751	-0.143 (-0.586 to 0.300)	0.526
General practice	0.111 (-0.571 to 0.794)	0.749	0.108 (-0.097 to 0.312)	0.302	-0.319 (-0.958 to 0.319)	0.327	-0.011 (-0.497 to 0.476)	0.965
Hospital	0.168 (-0.260 to 0.597)	0.442	-0.004 (-0.196 to 0.189)	0.969	0.135 (-0.621 to 0.891)	0.727	-0.183 (-0.675 to 0.310)	0.467
**Frequency of risk assessment**								
One-off single event	Reference		Reference		Reference		Reference	
Once every 5 years	0.060 (-0.493 to 0.614)	0.831	0.142 (-0.102 to 0.386)	0.254	0.427 (-0.396 to 1.249)	0.309	-0.034 (-0.498 to 0.430)	0.887
Once every year	-0.004 (-0.593 to 0.586)	0.990	0.017 (-0.178 to 0.212)	0.863	0.468 (-0.367 to 1.304)	0.272	0.119 (-0.316 to 0.553)	0.592
Continuously for 2 weeks	0.153 (-0.654 to 0.959)	0.710	-0.145 (-0.589 to 0.298)	0.520	-0.079 (-1.506 to 1.348)	0.914	0.246 (-0.754 to 1.245)	0.630
Constantly	0.221 (-0.459 to 0.902)	0.524	0.040 (-0.249 to 0.328)	0.787	0.161 (-0.973 to 1.295)	0.781	0.384 (-0.224 to 0.992)	0.216
**Accuracy**								
Specificity	**0.137 (0.098 to 0.175)**	**<0.001**	**0.018 (0.002 to 0.035)**	**0.030**	0.019 (-0.023 to 0.061)	0.382	**0.096 (0.065 to 0.126)**	**<0.001**
Sensitivity	**0.242 (0.182 to 0.301)**	**<0.001**	**0.022 (0.004 to 0.039)**	**0.017**	**0.044 (0.011 to 0.076)**	**0.009**	**0.147 (0.109 to 0.184)**	**<0.001**
**Class membership**								
Female sex (versus male)	0.003 (-0.479 to 0.485)	0.990	Reference		**-1.308 (-2.044 to -0.572)**	**<0.001**	**-0.809 (-1.382 to -0.235)**	**0.006**

10. Supplementary Table 8. Participants’ reported views on the relative acceptability of using cancer risk assessments in screening and referral contexts.

	Asymptomatic context cohort	Symptomatic context cohort	Total (%)
Total *N*	601	601	1,202 (100.0)
It is *more* acceptable to use a cancer risk assessment to decide *how much screening someone is offered*	67	68	135 (11.2)
It is more acceptable to use a cancer risk assessment to decide how urgently and thoroughly someone's symptoms are investigated	146	171	317 (26.4)
Both are *equally* acceptable	368	340	708 (58.9)
Neither are acceptable	20	22	42 (3.5)

P value for difference=0.364 (χ^2).

11. Supplementary Figure 3. Participants' ranking of the attributes of risk assessments.

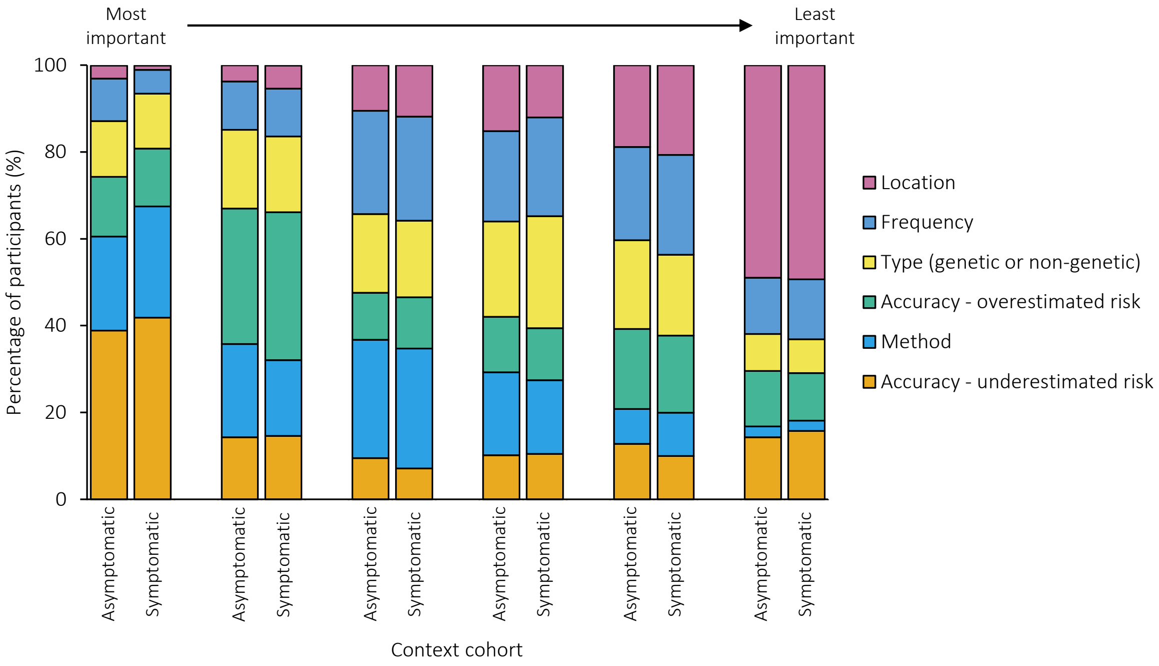

34 (2.8%) participants did not change the order of attributes from that presented in the question.
