# Supplementary material for: Risk-based innovations in cancer screening and diagnosis: a discrete choice experiment to explore priorities of the UK public
Source: BMJ Open. 2025 May 31;15(5):e093803. doi: 10.1136/bmjopen-2024-093803 (PMC12142028; doi:10.1136/bmjopen-2024-093803)
Supplement: Supplementary file 2 [file bmjopen-2024-093803.reviewer_comments.pdf]

PEER REVIEW HISTORY

BMJ Open publishes all reviews undertaken for accepted manuscripts. Reviewers are asked to complete a checklist review form and are provided with free text boxes to elaborate on their assessment. These free text comments are reproduced below.

ARTICLE DETAILS

Title (Provisional)

Risk-based innovations in cancer screening and diagnosis: a discrete choice experiment to explore the priorities of the UK public

Authors

Dennison, Rebecca; Morris, Stephen; Clune, Reanna J; Wright, Stuart; Waller, Jo; Usher-Smith, Juliet

---

VERSION 1 - REVIEW

**Reviewer**	**1**
**Name**	**Nabi, Hermann**
**Affiliation**	**Hôpital du Saint-Sacrement**
**Date**	**13-Nov-2024**
**COI**	**None**

The study explored public preferences for risk-based innovations in cancer screening and diagnosis through a discrete choice experiment (DCE) with 1,202 participants from the UK. The survey evaluated six attributes of risk assessment methods: test method, type (genetic vs. non-genetic), location, frequency, sensitivity, and specificity. Participants were asked to choose between different risk assessment options or opt out, with scenarios set in either asymptomatic or symptomatic contexts.

The authors found that participants generally preferred risk assessments over opting out, with sensitivity being the most important attribute, followed by test method and specificity. Genetic and non-invasive tests were particularly favored. When sensitivity and specificity were 80% or higher, the likelihood of opting for risk assessment increased. A majority of participants were very likely to participate in risk-based innovations, regardless of the context or method, while a smaller group would decide based on method, sensitivity, and specificity. Finally, a minority strongly opposed risk-based innovations, especially in screening settings.

There seems to be a strong public support for risk-based innovations in cancer healthcare, especially for referrals and using genetic or non-invasive tests. However, to increase

participation, it is important to optimize these innovations based on the test method and performance metrics, particularly for those whose interest depends on these factors.

This is an important topic, and the research is methodologically robust, with a relatively large sample that is representative of the UK population in terms of certain sociodemographic characteristics. However, it is worth noting that Discrete Choice Experiments (DCE) are complex and require significant participant engagement. As a result, over 25% of the sample did not complete the questionnaire correctly. The authors should discuss in more detail how this may have affected their findings.

Additionally, there were notable differences between the symptomatic and asymptomatic cohorts, and further discussion on these differences is needed.

In DCE studies, attribute development is typically informed by literature reviews, expert consultations, and qualitative interviews to ensure the attributes are relevant to participants. However, no qualitative research was conducted in this study, and the authors should provide a rationale for why this step was not included.

Finally, this manuscript is dense and some results are not easy to grab. The authors might consider simplifying it a little bit for the readership.

**Reviewer**	**2**
**Name**	**Melzer, , Anne C.**
**Affiliation**	**Minneapolis VA Health Care System**
**Date**	**18-Feb-2025**
**COI**	**None**

A number of additional risk prediction models and biomarkers are in development that will likely change or expand the way that cancer screening is provided. The authors present the results of a discrete choice experiment assessed via online survey. Using latent class analysis, they identified several latent classes that influence receptiveness to different types of risk prediction tests at varied frequencies and in varied locations. A strength of this paper is the consideration of public perception and acceptability of various risk-assessment strategies, as this is often considered late in the process of developing new tools. Failure to account for this may lead an otherwise promising tool to fail to be implemented.

Background:

Line 73-74: suggest making this two separate sentences for clarity

Methods:

Survey administration methods are strong including community input for readability and construction of the survey, randomization of symptomatic vs asymptomatic state and randomization of question order.

While not a true population weighted survey, sampling was completed in two blocks first representative of the demographics of the UK then across socioeconomic groups. Please clarify further how the recruitment took place. Was it entirely electronic? From clinics? This may influence the representativeness of the population.

The authors describe the process of latent class analysis well, but not as well what this 'means' for the unfamiliar reader. Please add some explanation of the 'uses' for latent class analysis.

Results:

The patterns for the choice of 'neither' are interesting. I'm not sure there is space but would consider moving some of this out of the supplement.

Please clarify how the Specificity/sensitivity coefficients should be interpreted in the table. It is in the footnotes but it is challenging to interpret. Perhaps just note in the table that the referent was placed at -12.5.

The section 'preferences for different aspects' focuses more on the between cohort differences. Considering that this type of analysis is not going to be familiar to much of the readership, I suggest adding some more context for the reader for some of these findings to make sense of the magnitude of the differences in preferences. Is there published literature that link hierarchy of preferences to health screening behaviors?

Preference against 'patient data-driven' risk assessments is very interesting as it runs counter to some common assumptions about 'ease' of testing. This has direct implications for proposed methods of determine screening eligibility, such as 'risk-based' lung cancer screening.

I recommend some formatting to make the interpretation of table 4 more clear. Perhaps bold or underline the relevant 'referent' in the interpretation note. It is more understandable in the text.

In general, there is a very large amount of data presented and much of it is in the supplement. This hinders the overall readability of the paper.

Discussion:

The latent class analysis is very interesting and will likely 'ring true' to many clinicians. The discussion does a fair job of making this clinically relevant. There is a lot of restating of results. Consider decreasing this and adding more public health implications or how this information could guide policymakers.

The limitations are well described.

Minor:

Line 178 I believe it should be 'analyze'

VERSION 1 - AUTHOR RESPONSE

Reviewer 1 Comments to the Author:

The study explored public preferences for risk-based innovations in cancer screening and diagnosis through a discrete choice experiment (DCE) with 1,202 participants from the UK. The survey evaluated six attributes of risk assessment methods: test method, type (genetic vs. non-genetic), location, frequency, sensitivity, and specificity. Participants were asked to choose between different risk assessment options or opt out, with scenarios set in either asymptomatic or symptomatic contexts.

The authors found that participants generally preferred risk assessments over opting out, with sensitivity being the most important attribute, followed by test method and specificity. Genetic and non-invasive tests were particularly favored. When sensitivity and specificity were 80% or higher, the likelihood of opting for risk assessment increased. A majority of participants were very likely to participate in risk-based innovations, regardless of the context or method, while a smaller group would decide based on method, sensitivity, and specificity. Finally, a minority strongly opposed risk-based innovations, especially in screening settings.

There seems to be a strong public support for risk-based innovations in cancer healthcare, especially for referrals and using genetic or non-invasive tests. However, to increase participation, it is important to optimize these innovations based on the test method and performance metrics, particularly for those whose interest depends on these factors.

This is an important topic, and the research is methodologically robust, with a relatively large sample that is representative of the UK population in terms of certain sociodemographic characteristics. However, it is worth noting that Discrete Choice Experiments (DCE) are complex and require significant participant engagement. As a result, over 25% of the sample did not complete the questionnaire correctly. The authors should discuss in more detail how this may have affected their findings.

RESPONSE: Thank you for the positive review of review of our research and its importance.

We believe this concern comes from the statement that '74% (883 participants) correctly answered all the questions to test their comprehension of the example table of risk assessment options (Supplementary Table 4)' (lines 194-195 in the original manuscript). Although it is true that DCEs require significant participant engagement, this does not mean *25% of the sample did not complete the questionnaire correctly*, but they got one or more of the comprehension questions wrong. We have revised the description and statistic reported to clarify this. We have also commented on this in the strengths and limitations of the discussion.

> "**All three of the questions designed to test comprehension of the table of risk assessment options, completed before the DCE exercise,** were answered correctly by 74% (883 participants), **with an average 90% of participants answering each question correctly** (Supplementary Table 4)." (Lines 212-216)

Additionally, there were notable differences between the symptomatic and asymptomatic cohorts, and further discussion on these differences is needed.

RESPONSE: The asymptomatic and symptomatic cohorts were very similar in terms of demographics and characteristics. We have added p values for the difference between cohorts (using Chi-squared tests) to Table 2 to demonstrate this. The only statistically significant difference between the cohorts is for personal history of cancer, which we have added a comment about.

> "Table 2 and Supplementary Table 5 show that the participants' demographic characteristics and thoughts and beliefs about cancer and screening were similar between cohorts; **the only statistically significant difference was that more participants in the asymptomatic context cohort had a personal history of cancer."** (Lines 218-221)

In DCE studies, attribute development is typically informed by literature reviews, expert consultations, and qualitative interviews to ensure the attributes are relevant to participants. However, no qualitative research was conducted in this study, and the authors should provide a rationale for why this step was not included.

RESPONSE: We have conducted qualitative research that informed attribute development, but had not described it in the paper. Thank you for highlighting this omission. We have added the following text, describing and citing the two paper that have been published since submitting this manuscript.

> "These attributes were informed by reviews of the literature, **our qualitative research that formed part of the same grant,** and discussion with experts in DCE design and PPI representatives. **The qualitative research was a set of three community juries (24 participants in total) and think-aloud interviews (21 participants) in which members of the public considered six different innovative approaches to be acceptable to assess risk of cancer[22,23]. The attributes were selected, therefore, to cover** a range of characteristics of risk assessments that **these studies showed** to impact public opinions and to enable us to compare preferences for different examples of risk-based innovations. **For example, along with test accuracy that was essential, and the effort/burden to take part was also important so we included test location and frequency attributes."** (Lines 124-133)

Finally, this manuscript is dense and some results are not easy to grab. The authors might consider simplifying it a little bit for the readership.

RESPONSE: We believe that the changes made in response to the peer reviews have substantially improved the understandability of this paper. This includes adding more explanation of the results in the text, adding new figures or moving them from the supplement to the main paper, and emphasising implications in the discussion.

Reviewer 2 Comments to the Author:

A number of additional risk prediction models and biomarkers are in development that will likely change or expand the way that cancer screening is provided. The authors present the results of a discrete choice experiment assessed via online survey. Using latent class analysis, they identified several latent classes that influence receptiveness to different types of risk prediction tests at varied frequencies and in varied locations. A strength of this paper is the consideration of public perception and acceptability of various risk-assessment strategies, as this is often considered late in the process of developing new tools. Failure to account for this may lead an otherwise promising tool to fail to be implemented.

RESPONSE: Many thanks for reviewing our paper.

Background: Line 73-74: suggest making this two separate sentences for clarity

RESPONSE: We have split this sentence into two as suggested.

> “Furthermore, these new risk-based technologies have the potential to move risk prediction away from the traditional healthcare context**. For instance, samples/data** could be collected in the home or continuously over a period of time, or through background analyses of routine medical records[11,13].” (Lines 74-76)

Methods:

Survey administration methods are strong including community input for readability and construction of the survey, randomization of symptomatic vs asymptomatic state and randomization of question order.

RESPONSE: Thank you for highlighting this.

While not a true population weighted survey, sampling was completed in two blocks first representative of the demographics of the UK then across socioeconomic groups. Please clarify further how the recruitment took place. Was it entirely electronic? From clinics? This may influence the representativeness of the population.

RESPONSE: All participants were recruited through the Prolific. We have added details about this recruitment platform that address these questions.

> “**Prolific is an online-only research recruitment platform with a pool of registered participants. It does not focus on health research, and has been found to result in the collection of higher quality data than comparable platforms[24]. … They were paid £2.50 for taking part.**” (Lines 148-159)

The authors describe the process of latent class analysis well, but not as well what this ‘means’ for the unfamiliar reader. Please add some explanation of the 'uses' for latent class analysis.

RESPONSE: We have added more explanation to the latent class analysis methods and added an illustrative example. We have also moved the more technical details to the end of the paragraph.

> “Latent class analysis was then used to divide the participants into groups with similar preferences **and priorities for risk assessments.** We then examined whether the classes **could be described by the participants’ characteristics (for example, whether female participants were statistically more likely to be in one class than another). The optimal number of groups, analysis seed and associations with binary characteristics were identified by examining the BIC (Supplementary Table 3b-d).”** (Lines 176-184)

Results:

The patterns for the choice of ‘neither’ are interesting. I’m not sure there is space but would consider moving some of this out of the supplement.

RESPONSE: As suggested, we have moved the figure showing the frequency of selecting “neither” from the supplement to the main paper (Figure 1, line 249).

Please clarify how the Specificity/sensitivity coefficients should be interpreted in the table. It is in the footnotes but it is challenging to interpret. Perhaps just note in the table that the referent was placed at -12.5.

RESPONSE: We believe that this point relates to Table 3. Upon reviewing this, we realise that the footnote "Accuracy levels for no risk assessment were fixed at 12.5 in the analysis" is not applicable to this table, so have removed it. As noted in the response to the point below, we have also added more descriptions of these results to increase understandability.

The section 'preferences for different aspects' focuses more on the between cohort differences. Considering that this type of analysis is not going to be familiar to much of the readership, I suggest adding some more context for the reader for some of these findings to make sense of the magnitude of the differences in preferences. Is there published literature that link hierarchy of preferences to health screening behaviors?

RESPONSE: We have expanded the descriptions of these results, with a less emphasis on the comparison between the cohorts. We also note the explanation in the footnote of Table 3 that "Positive coefficients indicate a preference for the specified level compared to the reference; negative coefficients indicate a preference for the reference compared to the specified level."

> "Method of risk assessment and accuracy significantly affected preferences in both cohorts **(Table 3). Blood tests and non-invasive tests were preferred over questionnaires or data access. Compared to questionnaires or data access, wearable devices were preferred in the symptomatic context, but less preferred in the asymptomatic context.** In both contexts, participants preferred risk assessments that more accurately estimated risk of cancer. **The importance of specificity was similar in the two contexts, with coefficients of 0.042 and 0.048 (p=0.262 for difference between cohorts).** It was more important to have higher sensitivity for people with symptoms than in those without symptoms **with coefficients of 0.081 and 0.059, respectively (p<0.001 for difference between cohorts).** There were no differences between context cohorts in preference for whether a test was genetic or non-genetic, location or frequency of repeating the test, except that it was seen as unfavourable for asymptomatic people to attend the hospital compared with doing a home-based test. **Participants in the asymptomatic and symptomatic contexts had different preferences across the models as a whole (p<0.001).**" (Lines 251-264)

Preference against 'patient data-driven' risk assessments is very interesting as it runs counter to some common assumptions about 'ease' of testing. This has direct implications for proposed methods of determine screening eligibility, such as 'risk-based' lung cancer screening.

RESPONSE: Yes, this is an interesting point. Our qualitative research suggests that while lower burden of testing is favoured, concepts like perceived trustworthiness and/or effectiveness of data-driven risk assessments are more important. Participants considered many ways in which routine data could be out of date or incorrect, whereas biological samples (e.g. blood and non-invasive tests) were "sophisticated and objective" (doi's 10.3389/fcacs.2025.1522609 and 10.1186/s12889-025-21996-x). We have added a comment on this in the discussion.

> **"Our qualitative research suggests that while lower burden of testing is favoured, concepts like perceived effectiveness and invention coherence are more important[23]."** (Lines 403-405)

I recommend some formatting to make the interpretation of table 4 more clear. Perhaps bold or underline the relevant 'referent' in the interpretation note. It is more understandable in the text.

RESPONSE: Instead of presenting this information in a table, we have replaced Table 4 with a figure to make it easier to interpret (Figure 3, line 288).

In general, there is a very large amount of data presented and much of it is in the supplement. This hinders the overall readability of the paper.

RESPONSE: We have considered the results of this paper carefully and made a number of changes to increase overall readability of the paper:

- Moved the figure for frequency of opting out from the supplement to the main paper (Figure 1, line 249).
- Described "Preferences for different aspects of risk assessments" more thoroughly in the text (lines 250-264).
- Added a simple figure for the results of the relative attribute importance (Figure 2, line 278).
- Replaced Table 4 with Figure 3 for the willingness to trade-off sensitivity and specificity of risk assessments (Figure 3, line 288).
- Moved the table for participants' reported views on the relative acceptability of using cancer risk assessments in screening and referral contexts from the supplement to the main paper (Table 4, line 381).

The supplement therefore only contains informative to expand on or justify the analysis methods (DCE question matrix, details of model selection process, and impact of changing the value of the accuracy attributes for the reference (no screening) programme) and results with less impact/importance for the key messages (e.g. further participant characteristics, sensitivity analyses, evaluation, and self-reported attribute ranking). However, we would be happy to move this into the main paper if requested.

Discussion:

The latent class analysis is very interesting and will likely 'ring true' to many clinicians. The discussion does a fair job of making this clinically relevant. There is a lot of restating of results. Consider decreasing this and adding more public health implications or how this information could guide policymakers.

RESPONSE: Thank you for these pointers, which we agree with. We have reviewed the discussion and sought to minimise repetition of the results whilst still making it clear which findings we are referring to. We have also added more implications, such as:

> "**Overall, this provides evidence to support policymakers in taking steps towards implementing such tests within screening and primary care.**" (Lines 393-394)

> "**This suggests that these priorities can be reflected by those developing and implementing these technologies.**" (Lines 405-406)

Other changes can be seen in the marked manuscript as there are too many to copy here.

The limitations are well described.

RESPONSE: Thank you for this comment.

Minor: Line 178 I believe it should be 'analyze'

RESPONSE: Thank you for highlighting this. We have corrected the typo.

Other changes made

- Table 3 (line 203) – coefficients for sensitivity and specificity have been corrected to positive values; i.e. participants prefer options with a higher sensitivity and specificity. (The previous negative values had been calculated within an earlier analysis when these were framed as over- and under-diagnosis.)
- Specification of the Delta method used to calculate the marginal rates of substitution (lines 188-189).

VERSION 2 - REVIEW

Reviewer 2

Name Melzer, , Anne C.

Affiliation Minneapolis VA Health Care System

Date 14-May-2025

COI

The authors present a revised manuscript describing the results of a discrete choice experiment assessed via online survey. Using latent class analysis, they identified several latent classes that influence receptiveness to different types of risk prediction tests for cancer at varied sensitivities, frequencies, formats, and in varied locations. They also identified key aspects of test performance that drive intention to participate in screening at all, primarily the sensitivity and specificity of the test.

The revised results are much more clear as they have substituted figures for several very complex tables. They have addressed my previous comments. in particular clarifying that this survey was based on a previous qualitative assessment.

A few minor comments:

Revised sentence starting line 128 is confusing as written, as is the one starting at 131

Sentence line 385 starting in “they” doesn’t seem grammatically correct.
